# 16S rRNA sequence dataset for the identification of the French hoverflies (Diptera, Syrphidae)

**DOI:** 10.3897/BDJ.14.e189822

**Published:** 2026-06-15

**Authors:** Anaïs Marquisseau, Mélodie Ollivier, Christophe Klopp, Géraldine Pascal, André Pornon, Nathalie Escaravage, Veronique Sarthou, Jean-Pierre Sarthou, Magalie Pichon

**Affiliations:** 1 Dynafor, INRAE, INP, ENSAT, 31326, Castanet-Tolosan, France Dynafor, INRAE, INP, ENSAT, 31326 Castanet-Tolosan France; 2 INP AgroToulouse, Av. de L’Agrobiopole, 31326, Auzeville-Tolosane, France INP AgroToulouse, Av. de L’Agrobiopole, 31326 Auzeville-Tolosane France; 3 University of Toulouse, Sigenae, GenoToul Bioinformatics Facility, BioinfOmics, INRAE, UR 875 MIAT, Castanet-Tolosan, France University of Toulouse, Sigenae, GenoToul Bioinformatics Facility, BioinfOmics, INRAE, UR 875 MIAT Castanet-Tolosan France; 4 GenPhySE, Université de Toulouse, INRAE, ENVT, 31326, Castanet-Tolosan, France GenPhySE, Université de Toulouse, INRAE, ENVT, 31326 Castanet-Tolosan France; 5 CBRE, CNRS-IRD-Toulouse INP-UT3, University of Toulouse, 31062, Toulouse cedex 09, France CBRE, CNRS-IRD-Toulouse INP-UT3, University of Toulouse, 31062 Toulouse cedex 09 France; 6 SYRPHYS Agro-Environnement, Le Soulas 630 C, Chemin du Moulin 31470, Bonrepos sur Aussonelle, France SYRPHYS Agro-Environnement, Le Soulas 630 C, Chemin du Moulin 31470 Bonrepos sur Aussonelle France

**Keywords:** biodiversity monitoring, DNA barcoding, natural enemies, pollinators, species molecular identification

## Abstract

With ongoing biodiversity loss, a wide range of taxa are being monitored to support the implementation of conservation measures aimed at preventing their decline. Effective monitoring requires the development of robust and scalable tools and methods. Amongst these, DNA barcoding and metabarcoding have emerged as powerful and widely adopted approaches for tracking biodiversity. To achieve high taxonomic resolution in molecular-based monitoring, the use of multiple DNA markers is often essential.

In this study, we focused on Syrphidae, a family that plays a key role in ecological networks. Alongside bees, hoverflies are remarkable pollinators within the order Diptera. Furthermore, larvae of the subfamily Syrphinae are widely regarded as beneficial in agricultural contexts, contributing to the biological control of pest populations. Mainland France, with its temperate climate, is a hotspot for Syrphidae diversity, hosting 566 species whose biological and ecological traits are thoroughly documented in the Syrph the Net 2024 database. France currently holds the highest recorded number of Syrphidae species in Europe.

We produced a set of 16S rRNA sequences for Syrphidae, well suited for environmental biomonitoring applications. A total of 713 specimens from the private collection of specialist entomologists, representing 352 species across 14 tribes, were sequenced. Using a customised validation workflow, 561 sequences covering 316 species were retained, representing approximately 60% of the French hoverfly fauna. For 166 species, the 16S rRNA marker successfully discriminates specimens at the species level, including morphologically challenging genera such as *Paragus*, *Melanostoma* and *Microdon*. For the remaining 150 species in the dataset, the 16S rRNA marker enables identification at the genus level, though species-level resolution would require additional specimens to be sequenced. This study represents a first step towards the development of a comprehensive multi-marker reference library for French Syrphidae.

## Introduction

Syrphidae, commonly known as hoverflies, are a diverse group of Diptera renowned for their hovering flight and their crucial roles within ecological networks. They provide a range of ecosystem services, most notably pollination (Dunn et al. 2020). Adult syrphids are considered the second most important group of pollinators of food crops after bees (Klecka et al. 2018, Lucas et al. 2018, Wotton et al. 2019, Doyle et al. 2020, Rader et al. 2020) and, in certain parts of northern Europe, they are even regarded as the most important pollinators of some culinary herbs (Toivonen et al. 2022) and fruit trees or shrubs (Brown and McNeil 2009, Davis et al. 2026), particularly under harsh climatic conditions. Larval stages of many Syrphidae species contribute to biological control, with 355 species identified as aphidophagous in Europe (Speight et al. 2024). Amongst them, *Episyrphus balteatus* is particularly prolific, capable of producing between 2,000 and 4,500 eggs over its lifetime (Branquart and Hemptinne 2000), which hatch into nocturnal larvae, specialised in aphid predation. In south-western France, up to seven generations of *E. balteatus* can occur annually. Syrphid adults display remarkable morphological diversity in size, shape and colouration (Bot and Van de Meutter 2023) and many species exhibit morphological and behavioural mimicry of bees and wasps (Howarth and Edmunds 2008), which can lead to misidentification by non-specialists. One of the most well-known examples of mimicry in Syrphidae is *Volucella bombylans*, which can imitate several species of bumblebees (Edmunds and Reader 2014). In general, species identification is carried out on adult specimens by specialists using morphological identification keys (Speight and Sarthou 2017, Speight et al. 2021). However, in certain taxa, adults cannot be reliably distinguished, based on morphology alone, necessitating additional information from larval stages or phenological and ecological data. This is particularly true for specialised groups such as *Microdon* (Speight 2024). Morphological identification becomes even more challenging in species complexes such as *Merodon* (Vujić et al. 2024) or in species exhibiting pronounced sexual dimorphism, as is the case in the genus *Sphaerophoria* (Gojković et al. 2020) and *Pipizella* (Sarthou et al. 2021).

Currently, nearly 6,300 species of Syrphidae are recognised worldwide, classified into four subfamilies (Microdontinae, Eristalinae, Syrphinae and Pipizinae) and sixteen tribes (Reemer 2013, Mengual et al. 2015, Mengual et al. 2022, Wu et al. 2024). In Europe, the Syrph the Net initiative (Speight et al. 2015, Speight 2020, Speight et al. 2024) has made significant contributions to the nomenclatural standardisation and ecological referencing of the 1025 hoverfly species currently recorded (Reverté et al. 2023). Of these, 37.2% (314 species) are considered threatened, based on the European Red List criteria, classified as Critically Endangered (CR), Endangered (EN) or Vulnerable (VU) (Vujić et al. 2022). Mainland France hosts the highest Syrphidae species richness in Europe, with 566 species documented (Speight et al. 2024), followed by Italy (513 species) and Switzerland (492 species) (Reverté et al. 2023). The distribution patterns of Syrphidae across European France, recently mapped by Speight et al. (2024), reveal high variability amongst species: while some are widely distributed across the country, others are restricted to specific geographic areas. For instance, *Cheilosia alpestris* has only been recorded at the Bretolet Pass in the Valais Alps (Aubert et al. 1976). This species, characteristic of the herbaceous layer of alpine meadows, is also known from Switzerland and Austria (Speight et al. 2024).

Recent advances in our understanding of phylogenetic relationships within the Syrphidae have been largely driven by phylogenomic studies combining traditional morphological analyses with modern molecular techniques (Wiegmann et al. 2011, Pauli et al. 2018, Wong et al. 2023). For example, Young et al. (2016) supported previous hypotheses of hoverfly phylogeny using anchored hybrid enrichment (AHE), a method that selectively targets and enriches specific genomic regions. More recently, Mengual et al. (2022) employed exon-capture sequencing in Syrphidae, demonstrating its utility for constructing a robust phylogenetic framework for further investigations into the evolution of mimicry, co-evolutionary dynamics between Microdontinae and their ant hosts, larval life history traits and the emergence of migratory behaviour. With the increasing accessibility of whole-genome sequencing (WGS), new opportunities for large-scale phylogenetic and evolutionary analyses have emerged. For instance, Wu et al. (2024) sequenced and analysed genomes from 25 species of Syrphidae across the subfamilies Eristalinae and Syrphinae, revealing that Syrphinae diversification temporally coincided with the rapid radiation of aphids. A significant contributor to the growing repository of Syrphidae genomic data is the Darwin Tree Project (Blaxter et al. 2022). These genomic data are particularly valuable, not only for resolving evolutionary relationships, but also for the development of effective primers for DNA barcoding and metabarcoding applications.

In recent years, barcoding and metabarcoding have gained considerable traction for monitoring biodiversity across a variety of contexts (Skevington et al. 2019, Buchner et al. 2024, Remmel et al. 2024, Salis et al. 2024). These approaches have demonstrated high efficiency, particularly in terms of rapid data acquisition and large-scale applicability (Ritter et al. 2019, Leandro et al. 2024). Such methods are especially well-suited for insect monitoring, a task that presents substantial challenges due to the immense taxonomic and functional diversity of this group (Buchner et al. 2024). Effective implementation of these techniques relies heavily on the availability of high-quality, curated reference databases, as well as the strategic selection of multiple DNA markers to maximise taxonomic resolution and coverage. In the case of hoverflies (Diptera, Syrphidae), DNA barcoding strategies have proven effective in uncovering cryptic diversity in multiple instances. For example, Rojo et al. (2006) conducted an in-depth taxonomic study of the West Palaearctic *Pandasyopthalmus* species (Paragini) by combining two molecular markers, COI and ITS2, with morphological analyses using MEB microscopy on both larval and adult stages. Additional barcoding studies have further contributed to regional taxonomic clarification: Jordaens et al. (2015) focused on Afrotropical Syrphidae, while Ståhls et al. (2009) applied molecular tools to delineate 22 *Merodon* species on Lesvos Island, Greece. In a broader context, Morinière et al. (2019) developed a comprehensive DNA barcode reference library for Diptera in Germany, based on COI sequences and encompassing 5,200 Barcode Index Numbers (BINs). The Barcode Index Number System is an analytical framework that algorithmically clusters sequences and assigns each cluster to its corresponding species. Despite achieving reliable species-level assignments in many cases, they observed low interspecific divergence within several dipteran families, including Syrphidae, Tachinidae and Calliphoridae. These findings suggest that the COI marker alone may be insufficient for accurate species discrimination in certain taxa and highlight the need for complementary markers to improve taxonomic resolution (Morinière et al. 2019). Similarly, the COI was an inefficient marker to identify the genus *Sphaerophoria* at the species level (Gojković et al. 2020).

The 250 bp 16S rRNA marker was described a few years ago as effective for insect barcoding and metabarcoding (Marquina et al. 2019) and has since been applied in eDNA approaches to monitor vertebrate diversity from airborne samples (Lynggaard et al. 2023) and to investigate pollinator communities using flower-derived material (Jones et al. 2025). This marker was previously employed in our laboratory to develop a 16S rRNA reference library for wild bees (Marquisseau et al. 2025) and the present dataset extends its application to Syrphidae, providing new reference sequences. We compiled available 16S rRNA and mitochondrial sequences of French Syrphidae from GenBank. We then produced 16S rRNA sequences from 561 specimens representing 316 species, sampled from the private collection of Jean-Pierre and Véronique Sarthou, specialists in hoverfly identification. Finally, we assessed the discriminative power of the 16S rRNA fragment in distinguishing species across Syrphidae tribes and genera. Genera were assigned to one of three discrimination categories, based on the 16S rRNA marker's ability to distinguish the species: full (100%), partial and none (0%). This dataset will facilitate DNA-based species identification within integrative taxonomy frameworks and metabarcoding applications.

## Methods

**Figure 1.**
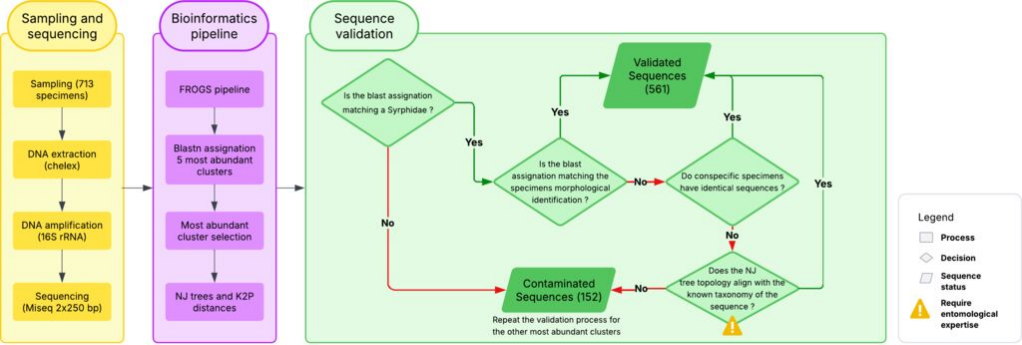
**Validated sequence production workflow: from specimen legs to final validated sequences**. The generation of validated sequences involves three main steps. The first step (yellow) covers the laboratory procedures required to obtain raw sequences. The second step (purple) consists of the bioinformatics pipeline, including the FROGS pipeline (Escudié et al. 2017), BLASTn assignment (Altschul et al. 1990, Camacho et al. 2009) and Neighbour-Joining (NJ) distance-tree inference using the K2P model (Kimura 1980). The third step (green) summarises the sequence validation process, based on the various criteria indicated by the diamond-shaped elements in the diagram.

We first conducted a systematic survey of 16S rRNA sequences already available in international databases for French Syrphidae. Then, we collected and sequenced specimens to build a French reference 16S rRNA dataset, following the workflow summarised in Fig. 1, which covers the full procedure from sampling to sequence validation.

### Extraction of French Syrphidae 16S rRNA and mitochondrial sequences from GenBank

French Syrphidae sequences were extracted from GenBank (Benson et al. 2012, 02-09-2025). Non-annotated mitochondrial sequences were retained only if their length exceeded 10,000 bp, corresponding to two-thirds of the complete mitochondrial sequence. We used Syrph the Net (Speight et al. 2024) as a checklist for French Syrphidae species.

### Sampling and sequencing

#### Sampling

The sampled specimens come from the Jean-Pierre and Véronique Sarthou collection. The specimens were captured in France with nets and killed by exposure to cyanide vapour. After identification, individuals were stored in Eppendorf tubes preserved in 70% ethanol and stored at room temperature. Three additional European species from the collection were also sampled and appear in the dataset: *Merodon caucasicus*, *Microdon mutabilis* and *Myolepta difformis*. A total of 713 specimens representing 352 species were processed. These species belong to three subfamilies: Eristalinae, Microdontinae and Syrphinae. They are classified into 75 genera and 14 tribes: Bacchini, Brachyopini, Callicerini, Cerioidini, Eristalini, Merodontini, Microdontini, Milesiini, Paragini, Pipizini, Rhingiini, Sericomyiini, Syrphini and Volucellini. Of the 352 species analysed in our study, 72 were represented by a single individual, 202 by two, 75 by three and three by four.

All of the specimens were morphologically identified using Veen (2010), Speight and Sarthou (2017) and Speight et al. (2021).

#### DNA extraction

DNA was extracted from an entire leg of specimens using the Chelex method (Casquet et al. 2011). Tissues were incubated overnight at 55°C in 100 µl of Chelex preparation (10%) containing 10 µl of proteinase K (600 mAU/ml). After centrifugation, the supernatant was removed and kept at -20°C.

#### DNA amplification

PCRs were performed as described in Marquisseau et al. (2025) with the 16S rRNA primers ins16S_1R/ins16S_1F (R: TRRGACGAGAAGACCCTATA; F: TCTTAATCCAACATCGAGGTC, Clarke et al. (2014)).

#### Miseq sequencing

Libraries were constructed as described in Marquisseau et al. (2025) and sequenced on a single run of an Illumina MiSeq (2 × 250 paired-end reads), using the NGS core facility at the Génopole Toulouse Midi-Pyrénées.

### Bioinformatics pipeline

Raw reads were merged using FLASH (Magoč and Salzberg 2011) and the most abundant sequence was selected for each sample. Raw reads were also processed using the FROGS pipeline (version 3.1, Escudié et al. (2017)) and the five most abundant clusters were kept for each sample. The six sequences per sample were compared to the GenBank nt database using BLASTn (Altschul et al. 1990, Camacho et al. 2009) and assigned the name of the closest species. Sequences were then aligned and distances calculated within the BOLD v.4 workbench (Ratnasingham and Hebert 2007, Ratnasingham et al. 2024) using KALIGN (Lassmann and Sonnhammer 2005) and the Kimura two-parameter model (Kimura 1980). Intraspecific distances correspond to the genetic distance within species. Nearest-neighbour distances correspond to the minimum interspecific distance for each species. Neighbour-Joining (NJ) trees were constructed within the BOLD v.4 workbench.

### Sequence validation

Clusters assigned to non-Syrphidae species were eliminated. If a cluster assignment matched their morphological identification, the sequence was validated for the specimen and the other clusters were discarded. Sequences were validated, based on their similarity to conspecific specimens and the topology of the tree. Validated sequence proportion and discriminative power of the 16S rRNA marker were calculated for the Syrphidae tribes and genera within our dataset. The validated species proportion corresponds to the proportion of species with at least one valid sequence, relative to the total number of sampled species. The discriminative power of the 16S rRNA marker was evaluated with the genetic distances between species. The 16S rRNA marker was considered discriminant for a species when the maximum intraspecific genetic distance was lower than the distance to the nearest neighbour.

## Data Resources

The metadata can be found in Suppl. material 1 and the sequences in Suppl. material 2.

### Resource 1

Download URL: http://doi.org/10.5883/DS-BCSY16S 

Resource identifier: DS-BCSY16S

Data format : FASTA

## Additional information

This section provides insights into the dataset and sequences generated in this study. We first retrieved 16S rRNA and mitochondrial sequences of French Syrphidae species from GenBank, then assessed the discriminatory power of the 16S rRNA fragment in distinguishing species across Syrphidae tribes and genera. Genera were assigned to one of three species-level discrimination categories: full discrimination (100%), variable discrimination and no discrimination (0%).

Additional information

### 16S rRNA and mitochondrial sequences of French Syrphidae available in GenBank

**Figure 2.**
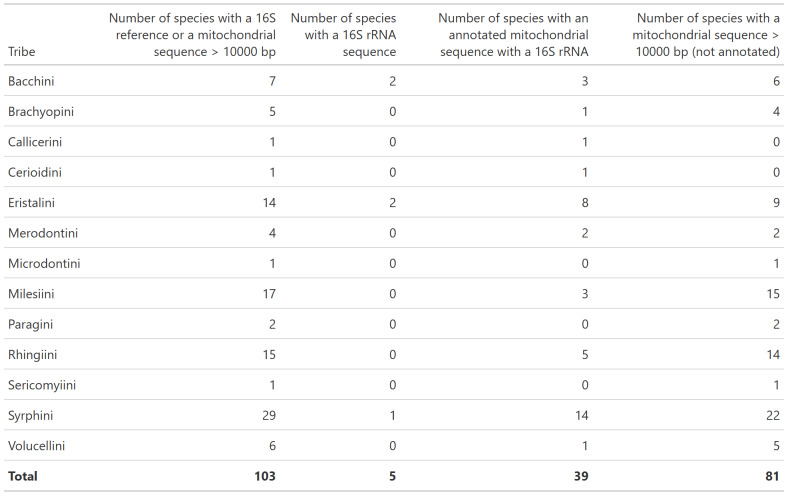
Number of French Syrphidae species with a 16S rRNA or mitochondrial sequence available in GenBank.

Of the 581 French species listed in Syrph the Net, 103 have at least one 16S rRNA sequence available in GenBank, originating from specimens collected outside France. Most of these sequences derive from mitochondrial genome sequencing projects, including 70 generated by the Darwin Tree of Life Project (Blaxter et al. 2022). Of the available mitogenomes, 39 are annotated, allowing direct access to the 16S rRNA gene, while 81 remain unannotated (Fig. 2). The full-length 16S rRNA gene ranges in size from 1,127 to 1,414 bp (Suppl. material 3). Five species (*Eristalis tenax*, *Eristalinus punctulatus*, *Melanostoma scalare*, *Melanostoma apicale* and *Cheilosia albitarsis*) have partial 16S rRNA sequences with sizes ranging from 369 to 1,079 bp.

Several species have multiple 16S rRNA reference sequences available in GenBank, such as *Episyrphus balteatus* and *Syritta pipiens*, each represented by five sequences. The tribe Syrphini is the best represented, with 29 species having at least one 16S rRNA reference sequence. In contrast, four tribes (Callicerini, Cerioidini, Microdontini and Sericomyiini) are each represented by a single reference sequence. Of the 352 species sampled in our work, 77 already had mitochondrial sequences available in GenBank before this study.

### Validated species proportion and species-level discriminative power of the 16S rRNA marker per tribe

**Figure 3.**
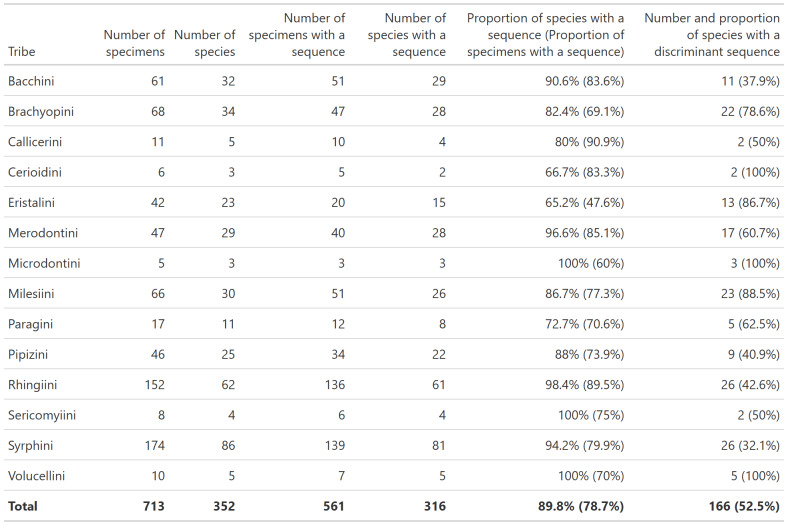
**Validated species proportion and species-level discriminative power of the 16S rRNA marker per tribe**. The validated species proportion corresponds to the proportion of species with at least one valid sequence, relative to the total number of sampled species. The discriminative power of the 16S rRNA marker was evaluated with the genetic distance between species. A species was considered discriminant when the intraspecific genetic distance was lower than the distance to the nearest neighbour. These genetic distances for all species in the dataset are provided in Suppl. material 6.

Our dataset comprises 713 specimens representing 352 species distributed across 14 tribes. The most species-rich tribes are Syrphini and Rhingiini, with 86 species (174 specimens) and 62 species (152 specimens), respectively. After removing contaminated sequences, identified with the aid of tree topology (Fig. 1, Suppl. material 4), a total of 316 species were retained for subsequent analyses. Validated species proportion ranged from 65% to 100%, with eleven tribes exceeding 80% validation (Fig. 3). For 36 species (Suppl. material 5), no valid sequence could be obtained.

The 16S rRNA marker successfully discriminated all species within the tribes Volucellini, Microdontini and Cerioidini. Notably, the tribes Bacchini and Brachyopini contain a similar number of species with at least one valid sequence (29 and 28 species, respectively), but the discriminative power of the 16S rRNA marker was twice as high for the tribe Brachyopini (79%) as for the tribe Bacchini (38%). The tribes Eristalini and Milesiini showed a particularly high proportion of discriminated species, at 89% and 87%, respectively.

### Species-level discriminative power of the 16S rRNA marker per genus

#### Genera for which the 16S rRNA marker is fully discriminant

**Figure 4.**
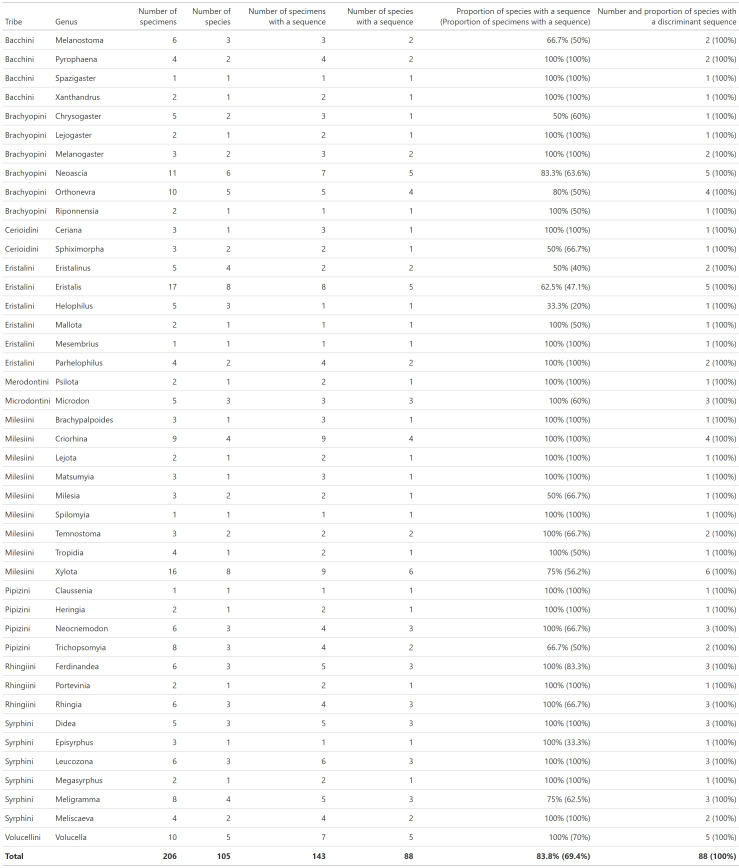
List of genera for which the 16S rRNA marker is fully discriminant.

Amongst the 75 genera included in our dataset, the 16S rRNA marker achieved 100% species-level discriminative power for 43 genera, successfully distinguishing all congeneric species in 21 cases (Fig. 4). For the 22 genera represented by a single species, validation was achieved through the application of multiple criteria from our workflow (Fig. 1), providing an initial set of reference sequences for specimens previously identified by entomologists. In the following section, we detail the six genera with the highest species and specimen numbers successfully barcoded: *Neoascia* (Brachyopini), *Eristalis* (Eristalini), *Xylota* (Milesiini), *Volucella* (Volucellini) and two genera for which identification at the species level is particularly challenging: *Melanostoma* (Bacchini) and *Microdon* (Microdontini).

**Neoascia**

The genus *Neoascia* comprises hoverflies typically associated with wetland habitats. Their larvae are semi-aquatic and adults are generally observed flying slowly amongst low-growing flowers in these environments. The genus has been primarily documented in Britain and Europe (Bartsch et al. 2009, Bot and Van de Meutter 2023), with additional records from Southeast Asia (Reemer and Hippa 2005) and North America (Skevington et al. 2019). In France, eight *Neoascia* species have been formally described (Speight et al. 2024), one of which, *Neoscia interrupta*, is classified as Endangered (EN) on the European Red List (Vujić et al. 2022). All eight species were included in the present study. The overall sequence validation rate reached 83% and the 16S rRNA marker successfully discriminated five of the eight species. Molecular data for this genus remain scarce, with available information limited to the complete mitochondrial genome of *N. interrupta* (Falk and Woodcock 2023) and COI sequences for 18 species, none of which originated from France.

Eristalis

The genus Eristalis comprises twenty-one species in Europe, fourteen of which are present in France (Speight et al. 2024). It belongs to the subfamily Eristalinae and the tribe Eristalini. Most *Eristalis* species mimic the western honey bee *Apis mellifera* and are commonly referred to as "droneflies", while others display a bumblebee-like appearance, such as *Eristalis nemorum* and *Eristalis intricaria* (Schulten 2019, Bot and Van de Meutter 2023). This mimicry also involves behavioural and acoustic mechanisms, including flight patterns and defensive buzzing (Golding et al. 2001, Moore and Hassall 2016). Their aquatic and saprophagous larvae, commonly known as "rat-tailed maggots", contribute to the decomposition of organic matter and to water and sediment purification. *Eristalis* larvae can accidentally develop in the digestive tract of mammals, including humans, leading to rare cases of intestinal myiasis reported, for example, in Palestine (Basha 2023), Brazil (Peruzzo et al. 2023) and France (Raffray and Malvy 2014). Amongst the genus, *Eristalis tenax* is the most widespread hoverfly species in the world and is frequently used as a model species for hoverflies. It can also act as a vector of bee parasites through visits to floral resources shared with bees (Davis et al. 2021). Consequently, *E. tenax* highest number of mitochondrial sequences available amongst French hoverfly species, followed closely by *Episyrphus balteatus*. Three additional species have had their mitochondrial genomes sequenced: *Eristalis arbustorum*, *E. intricaria* and *Eristalis pertinax* (Suppl. material 3) . Of the five Eristalis species sequenced using our 16S rRNA marker, represented by eight specimens, three had a mitochondrial sequence available in GenBank, matching our data with 100% identity. The 16S rRNA marker successfully discriminated all species both within our dataset and across sequences retrieved from GenBank.

Xylota

The genus *Xylota* belongs to the tribe Xylotini (also referred to as Milesiini). As the largest genus within the subtribe Xylotina, more than 130 *Xylota* species are currently recognised worldwide, with the highest diversity recorded in Northeast Asia, where 36 species have been documented (Jeong and Han 2019). In France, 11 species have been formally described (Speight et al. 2024), of which eight species (16 specimens) were sequenced in the present study. The validated species proportion was 75% and no sequence could be obtained for *Xylota albiens* and *Xylota migeniana*. For the remaining six species, the 16S rRNA marker achieved 100% discriminative power. *Xylota sylvarum* and *Xylota segnis* occur in nearly all French administrative Departments (*départements*). *X. sylvarum* is also widespread and abundant throughout the United Kingdom and continental Europe (GBIF Secretariat 2023) and is currently listed as "Least Concern" (Vujić et al. 2022). Like most Xylotini, *X. sylvarum* inhabits dense forested environments and can easily be confused with *Xylota xanthocnema* (Crowley and Nash 2023). The full-length mitochondrial sequences of *X. sylvarum* (Crowley and Nash 2023) and *X. segnis* are now available as direct submissions from the Darwin Tree of Life Project 2023. More recently, Ji et al. (2025) sequenced seven mitochondrial genomes from Asian taxa to re-evaluate Xylotini taxonomy and investigate mitochondrial genome evolution in hoverflies.

Volucella

The genus Volucella belongs to the subfamily Eristalinae and the tribe Volucellini. It is characterised by the plumose arista that gave it its common name: Plumehorn. Europe is home to six *Volucella* species, five of which can be found in France (Speight et al. 2024). *Volucella* species are commonly used as a textbook example of Bayesian mimicry, whereby their resemblance to Hymenoptera, such as bumblebees, wasps and hornets affords them protection from insectivorous birds. This resemblance is often specialised to match local Hymenoptera populations, as illustrated by the distinct morphs of *Volucella bombylans*, each mimicking different bumblebee species (Edmunds and Reader 2014). In four of the five French species, females lay their eggs in the nests of eusocial bees, wasps or hornets, where the larvae feed on nest debris or dead larvae. The exception is *Volucella inflata*, whose larvae develop in sap runs or insect tunnels in broad-leaved trees (Schulten 2019, Bot and Van de Meutter 2023). Complete mitochondrial sequences are available in GenBank for all five French species. All seven of our sequences matched the corresponding species when queried against the nt database using BLASTn (Suppl. material 3), with 100% identity and coverage, except for our *V. bombylans* specimen, which showed 98.62% identity. This discrepancy may reflect either geographic divergence, as the *V. bombylans* sequence in GenBank originates from the UK or intraspecific morphological variation. Further sequencing of additional specimens is needed to clarify this. Although the 16S rRNA marker effectively discriminates amongst the five species in our dataset, *Volucella pellucens* shares the same sequence with *Volucella latifasciata*, a non-European species, in GenBank (NC_068614.1). This overlap underscores the importance of developing local barcoding and metabarcoding databases, as well as the need for checklists supported by fieldwork and expert validation.

Melanostoma

The *Melanostoma* genus belongs to the tribe Bacchini. With seven species formally described in France, morphological identification is particularly challenging, especially for females. The entirely black thorax and elongated abdomen make confusion possible not only amongst *Melanostoma*, but also with certain *Platycheirus* species. The diagnostic character used to separate this genus from other Bacchini is the presence of an excavated metasternum (Andersson 1970, Barkalov 2009). This morphological distinction has been confirmed genetically, with phylogenetic studies placing these two genera in separate clades (Mengual et al. 2008, Mengual et al. 2015, Young et al. 2016, Mengual et al. 2022). Species-level identification is equally challenging using molecular approaches. Ståhls and Haarto (2014) demonstrated that COI does not reliably resolve species within *Melanostoma* and all German species were assigned to the same BIN (Morinière et al. 2019). In the Barcode of Life Data System (BOLD), a total of 2,334 specimens belonging to the genus *Melanostoma* have been recorded, of which 1,612 were assigned to unidentified species. Additionally, 2,244 COI sequences and four 16S rRNA sequences are currently available (October 2025). In the present study, we generated sequences for three specimens belonging to two species: *Melanostoma certum* and *Melanostoma scalare*. The two *M. certum* sequences were divergent from *M. scalare* by 0.93% and also distinct from the three *M. mellinum* 16S rRNA sequences available in BOLD.

*Microdon*

The genus *Microdon* presents particular challenges for entomologists, as its biology and ecology differ markedly from those of other Diptera. These myrmecophilous hoverflies spend part of their life cycle concealed within ant nests; their larval morphology closely resembles that of molluscan larvae and adults are morphologically similar to one another, displaying a bee-like appearance. The taxonomy of this genus has been extensively documented by Reemer (2013), Reemer and Ståhls (2013a) and Reemer and Ståhls (2013b). Long considered a distinct family (Speight 1994), Microdontidae has since been classified as a sister clade to all other hoverflies, based on molecular phylogenetic analyses (Young et al. 2016). A total of 249 species have been formally described worldwide, with the greatest diversity recorded in tropical regions. In Europe, the genus is more restricted, with only six species recognised. In France, five species have been recorded: three of them, *Microdon analis*, *Microdon devius* and *Microdon myrmicae*, are present across several French administrative Departments, while *Microdon miki* and *Microdon major* have each been recorded from a single French administrative Department (Speight et al. 2024). In the present study, sequences were generated for five specimens belonging to three species: *M. analis* (2 specimens), *M. miki* (1 specimen) and *Microdon mutabilis* (2 specimens). Following the validation workflow, only one sequence was retained for each of *M. miki* and *M. mutabilis*.

#### Genera for which the 16S rRNA marker discriminative power is variable

**Figure 5.**
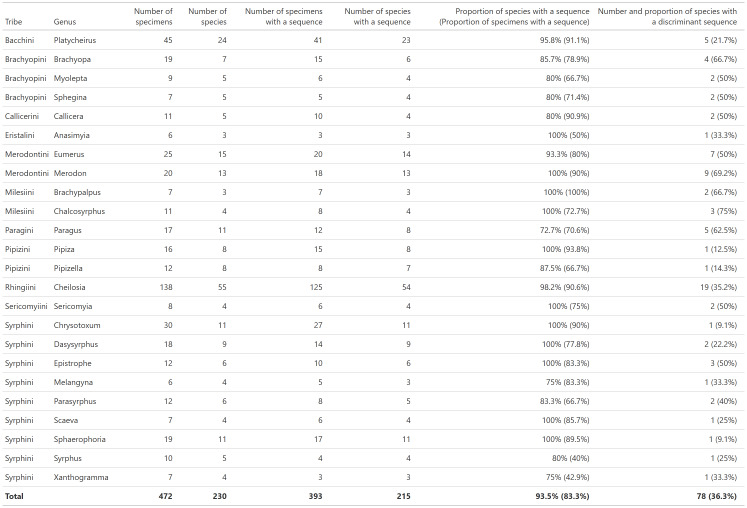
List of genera for which the 16S rRNA marker discriminative power is variable.

For 24 genera in our dataset, the discriminative power of the 16S rRNA marker varies substantially (Fig. 5). It is relatively high for some genera, reaching 86% for *Xylota* and 80% for *Eristalis*, but falls below 20% for four others: *Sphaerophoria* (18%), *Chrysotoxum* (10%), *Pipiza* (12%) and *Pipizella* (14%). In the following section, we provide a detailed examination of two genera: *Cheilosia*, which is represented by the largest number of specimens (138) and species (55) of our dataset and *Paragus*, for which morphological identification is particularly challenging and for which DNA barcoding could offer significant advantages.

*Cheilosia*

The genus *Cheilosia* is one of the most diverse and species-rich genera within the order Diptera, comprising approximately 445 species worldwide, 126 in Europe and 70 in France (Speight et al. 2024). This high diversity, combined with an adult phenotype ranging from small to large body sizes and predominantly black colouration, makes species-level identification particularly challenging. Furthermore, differences in nomenclature between Europe and Asia have led to extensive synonymy and the taxonomy continues to evolve through both morphological and molecular approaches. The molecular phylogenetic study of Rhingiini by Vujić et al. (2018), based on three markers (COI and nuclear 28S and 18S rDNA), clearly showed that the genus *Cheilosia* was monophyletic and distinct from *Rhingia*. Similarly, Ståhls et al. (2004) conducted a phylogenetic study of the genus and highlighted that relationships amongst species remained ambiguous, based on COI alone. More recently, Barkalov and Ståhls (2022) reported an inventory of 37 *Cheilosia* species classified with the COI barcode from the central and western region of Nepal and suggested that additional undescribed species remain to be discovered in the Himalayan Region. In the present study, sequences were generated for approximately 70% of French *Cheilosia* species, representing 55 species across 138 specimens. A search of international databases revealed that mitochondrial sequences were available for *Cheilosia albitarsis*, *Cheilosia grossa*, *Cheilosia impressa*, *Cheilosia pagana*, *Cheilosia scutellata*, *Cheilosia soror* and *Cheilosia vulpina*. The 16S rRNA marker successfully discriminated 24 species.

*Paragus*

The genus *Paragus*, comprising nearly one hundred described species worldwide, is the sole representative of the tribe Paragini. It is divided into two subgenera: *Paragus* and *Pandasyopthalmus* (Pape and Thompson 2009). In Spain, Paragus is the most species-rich genus of hoverflies, with 18 known species (Ricarte et al. 2017), while 21 species have been recorded in metropolitan France (Speight et al. 2024). These species are particularly difficult to identify due to high intraspecific variability in abdominal colouration, which ranges from black to yellow or orange depending on geographic location. Furthermore, females are morphologically indistinguishable from one another, which frequently leads to misidentification. A full-length mitochondrial genome sequence of *Paragus haemorrhous* was sequenced and deposited in worldwide databases as part of the Tree of Life project. Using both morphological and molecular characters, Vujic et al. (2008) reconstructed the phylogeny of 24 Paragini species using COI and 28S rRNA markers, proposing a revised classification comprising four subgenera rather than two. In the present study, sequences were generated for eight species representing 17 specimens recorded in France: *Paragus bicolor*, *Paragus quadrifasciatus*, *Paragus flammeus*, *Paragus albifrons*, *Paragus bradescui*, *Paragus pecchiolii*, *Paragus punctulastus* and *Paragus stigatus*. The 16S rRNA marker successfully discriminated five of the eight species. Additionally, variable sites were observed within the sequences of conspecific specimens of *P. bicolor*, *P. quadrifasciatus* and *P. flammeus* (data not shown).

#### Genera for which the 16S rRNA marker is not discriminative

**Figure 6.**
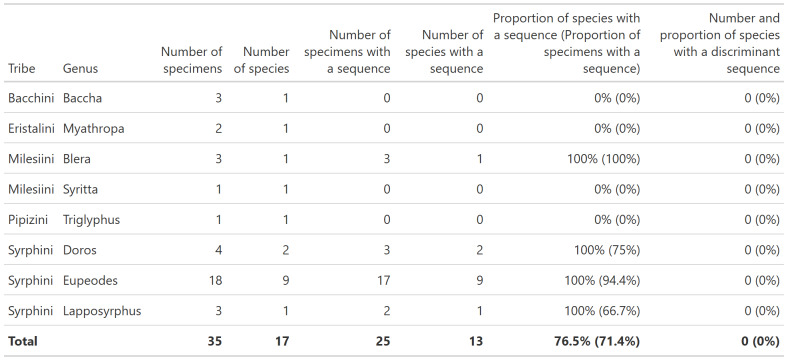
List of genera for which the 16S rRNA marker is not discriminative.

The discriminative potential of the 16S rRNA marker was not assessed for four genera without any validated sequences (*Baccha*, *Myathropa*, *Syritta* and *Triglyphus*). For the remaining four tribes (*Blera*, *Doros*, *Eupeodes* and *Lapposyrphus*), the 16S rRNA marker failed to discriminate conspecific specimens (Fig. 6). Below, we describe the genus with the highest species and specimens of this category.

*Eupeodes*

The genus *Eupeodes* is common and globally distributed, with many species exhibiting migratory behaviour (Wotton et al. 2019). Members of this genus occupy a wide range of habitats, including agricultural fields, orchards and meadows (Speight 2020). The genus comprises both banded and spotted species, which are often morphologically similar to other genera within the tribe Syrphini, making species-level identification challenging. *Eupeodes corollae* is the most studied species due to its ecological importance as both a pollinator and a natural enemy of aphids (Jiang et al. 2022, Li et al. 2023). Before our study, mitochondrial genome sequences were available for five species: *Eupeodes luniger*, *E. corollae*, *Eupeodes americanus*, *Eupeodes latifasciatus* and *Eupeodes confrater*. In total, 23 *Eupeodes* species have been formally described in Europe (Vujić et al. 2022), including 11 from France. In the present study, we provided sequences for nine species: *Eupeodes bucculatus*, *E. luniger*, *E. corollae*, *E. latifasciatus*, *Eupeodes flavipes*, *Eupeodes lucasi*, *Eupeodes nielseni*, *Eupeodes nitens* and *Eupeodes tirolensis*. The 16S rRNA marker failed to discriminate any of the nine species.

### Concluding Remarks

**Figure 7.**
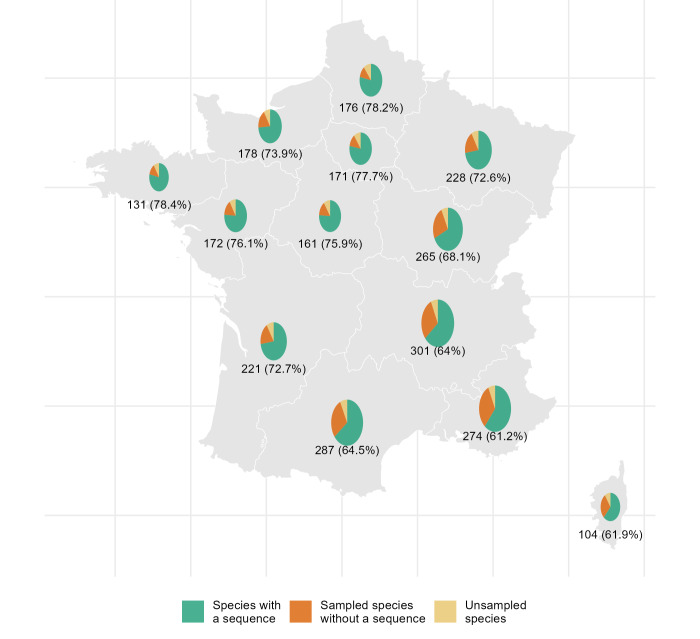
**16S rRNA dataset synthesis: Sampling effort and sequence coverage across French regions.** Values shown below each pie chart represent the total number of species for which at least one sequence was validated in this study and their proportion (green) relative to the total number of species recorded per French region according to Syrph the Net (Speight et al. 2024). Orange: Species sampled for which no valid sequence was obtained. Yellow: French species that were not sampled. *Pipiza fenestrata* specimens were not included in the figure because the nomenclature of the species has been revised into two species, *Pipiza noctiluca* and *Pipiza fasciata* and were not re-observed.

Like many other pollinator groups, hoverfly diversity is declining. Nevertheless, their high species richness and migratory capacity may confer a degree of resilience to ongoing environmental change. Although a European Red List for Syrphidae is available, no equivalent assessment has yet been established for France. Comprehensive inventories of Syrphidae remain challenging due to the limited number of specialist entomologists and the taxonomic difficulty of identifying certain tribes at the species level, as many adults display highly similar morphologies. In this study, we employed a fragment of the 16S rRNA gene to generate reference sequences for 561 Syrphidae specimens collected across France, encompassing 316 species. This dataset represents approximately 60% of the national fauna, with notable regional variation: the three south-eastern regions (Occitanie, Provence-Alpes-Côte d’Azur and Auvergne-Rhône-Alpes) emerge as major hotspots of Syrphidae diversity (Fig. 7, Suppl. material 7). Sampling efforts and molecular marker sequencing should, therefore, prioritise these regions in the future to complete the dataset.

## Supplementary Material

FDC855E4-1D86-578E-A2B1-787A31B0E6EE 10.3897/BDJ.14.e189822.suppl1 Supplementary material 1 Dataset Metadata Data type tsv File: oo_1625554.tsv https://binary.pensoft.net/file/1625554 Anaïs Marquisseau, Mélodie Ollivier, Christophe Klopp, Géraldine Pascal, André Pornon, Nathalie Escaravage, Jean-Pierre Sarthou, Véronique Sarthou, Magalie Pichon

5D2EF4EB-705C-517D-ACFE-E9762F5BA4CC 10.3897/BDJ.14.e189822.suppl2 Supplementary material 2 Dataset Sequences Data type fasta File: oo_1544668.fasta https://binary.pensoft.net/file/1544668 Anaïs Marquisseau, Mélodie Ollivier, Christophe Klopp, Géraldine Pascal, André Pornon, Nathalie Escaravage, Jean-Pierre Sarthou, Véronique Sarthou, Magalie Pichon

9255052B-8C99-5AD0-8BA3-7381CA3A35EA 10.3897/BDJ.14.e189822.suppl3 Supplementary material 3 List of mitochondrial or 16S rRNA sequences available in GenBank for French hoverfly species Data type tsv File: oo_1625569.tsv https://binary.pensoft.net/file/1625569 Anaïs Marquisseau, Mélodie Ollivier, Christophe Klopp, Géraldine Pascal, André Pornon, Nathalie Escaravage, Jean-Pierre Sarthou, Véronique Sarthou, Magalie Pichon

94D1FE6F-07B5-50A1-B5D8-332D4868CE2C 10.3897/BDJ.14.e189822.suppl4 Supplementary material 4 Neighbour-Joining Tree Data type NJ Tree File: oo_1625037.pdf https://binary.pensoft.net/file/1625037 Anaïs Marquisseau, Mélodie Ollivier, Christophe Klopp, Géraldine Pascal, André Pornon, Nathalie Escaravage, Jean-Pierre Sarthou, Véronique Sarthou, Magalie Pichon

140FB94B-1A8A-528B-AC6F-6F695868F414 10.3897/BDJ.14.e189822.suppl5 Supplementary material 5 List of sampled species with no validated sequence Data type tsv File: oo_1625574.tsv https://binary.pensoft.net/file/1625574 Anaïs Marquisseau, Mélodie Ollivier, Christophe Klopp, Géraldine Pascal, André Pornon, Nathalie Escaravage, Jean-Pierre Sarthou, Véronique Sarthou, Magalie Pichon

854B522B-BABB-5E16-A71E-E3EAB86AF7BA 10.3897/BDJ.14.e189822.suppl6 Supplementary material 6 Genetic distances Data type tsv File: oo_1625571.tsv https://binary.pensoft.net/file/1625571 Anaïs Marquisseau, Mélodie Ollivier, Christophe Klopp, Géraldine Pascal, André Pornon, Nathalie Escaravage, Jean-Pierre Sarthou, Véronique Sarthou, Magalie Pichon

A5BB7857-2D6E-5ECE-997C-014515EE726B 10.3897/BDJ.14.e189822.suppl7 Supplementary material 7 Summary of validated French Syrphidae sequences per French administrative Department (*département*) Data type tsv Brief description The first column lists the French Syrphidae species. Columns 2 to 7 provide species-level metrics: number of sampled specimens, number of validated sequences, presence in Syrph the Net (StN), availability of a 16S rRNA sequence generated in this study, availability of a mitochondrial sequence in GenBank prior to this study and availability of a 16S rRNA sequence in GenBank prior to this study. Columns 8 onwards indicate species presence (1) or absence (NA) across French Départements, identified by their codes. File: oo_1625577.tsv https://binary.pensoft.net/file/1625577 Anaïs Marquisseau, Mélodie Ollivier, Christophe Klopp, Géraldine Pascal, André Pornon, Nathalie Escaravage, Jean-Pierre Sarthou, Véronique Sarthou, Magalie Pichon
