# Supplementary material for: 16S rRNA sequence dataset for the identification of the French hoverflies (Diptera, Syrphidae)
Source: Biodivers Data J. 2026 Jun 15;14:e189822. doi: 10.3897/BDJ.14.e189822 (PMC13288028; doi:10.3897/BDJ.14.e189822)
Supplement: Supplementary material 4 — Neighbour-Joining Tree [file bdj-14-e189822-s004.pdf]

BOLD TaxonID Tree

Title	:	Tree Result - DS-BCSY16S (561 records selected)
Date	:	04-May-2026
Data Type	:	Nucleotide
Distance Model	:	Kimura 2 Parameter
Marker	:	16S
Colourization	:	Taxonomy: Tribe

Label	:	Sample ID
Label	:	Tribe
Label	:	Taxon

Sequence Count	:	561
Species count	:	316
Genus count	:	71
Family count	:	1
Unidentified	:	0

BIN Count	:	0

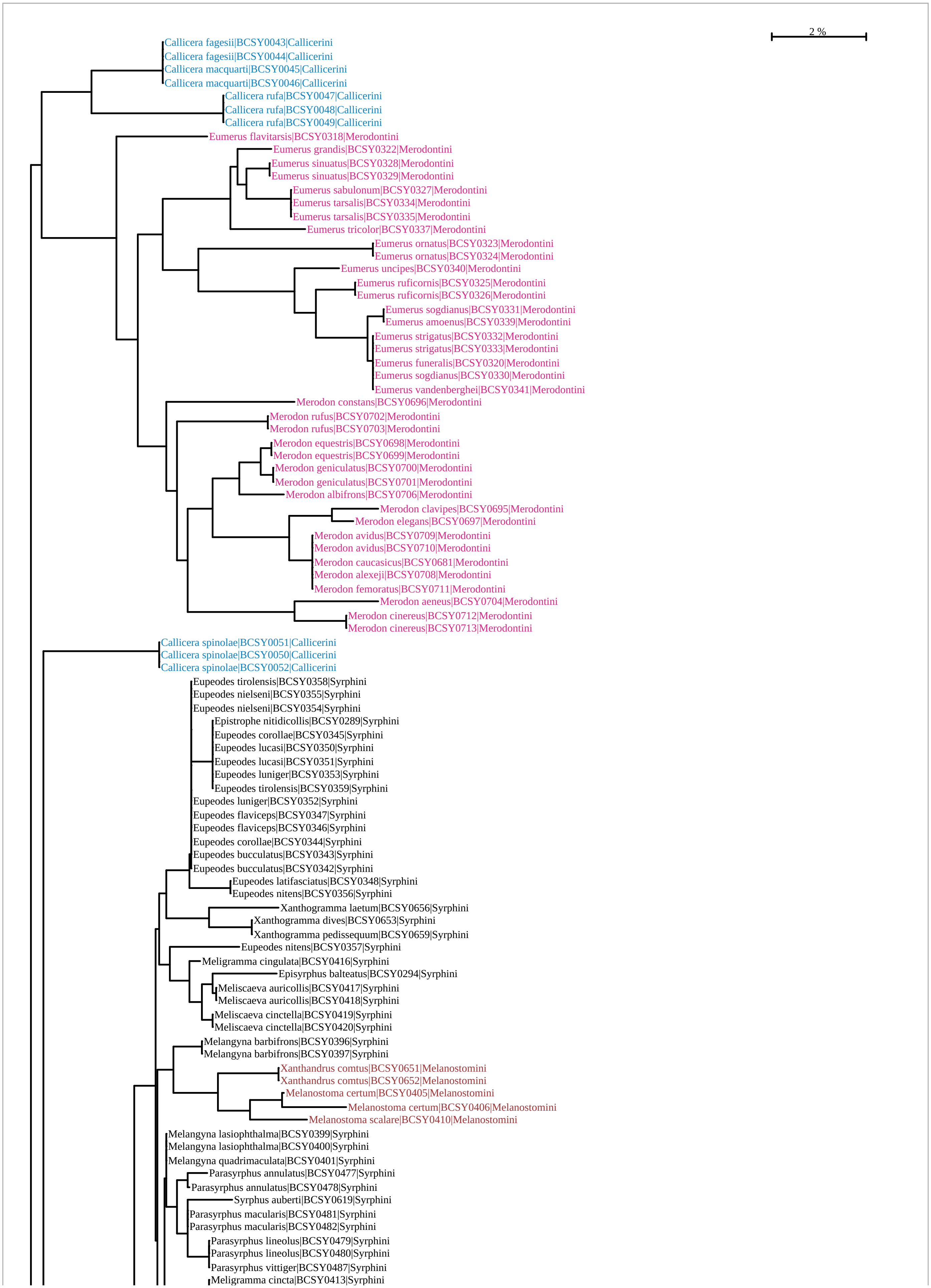
2 %
Callicera fagesii|BCSY0043|Callicerini
Callicera fagesii|BCSY0044|Callicerini
Callicera macquarti|BCSY0045|Callicerini
Callicera macquarti|BCSY0046|Callicerini
Callicera rufa|BCSY0047|Callicerini
Callicera rufa|BCSY0048|Callicerini
Callicera rufa|BCSY0049|Callicerini
Eumerus flavitarsis|BCSY0318|Merodontini
Eumerus grandis|BCSY0322|Merodontini
Eumerus sinuatus|BCSY0328|Merodontini
Eumerus sinuatus|BCSY0329|Merodontini
Eumerus sabulonum|BCSY0327|Merodontini
Eumerus tarsalis|BCSY0334|Merodontini
Eumerus tarsalis|BCSY0335|Merodontini
Eumerus tricolor|BCSY0337|Merodontini
Eumerus ornatus|BCSY0323|Merodontini
Eumerus ornatus|BCSY0324|Merodontini
Eumerus uncipes|BCSY0340|Merodontini
Eumerus ruficornis|BCSY0325|Merodontini
Eumerus ruficornis|BCSY0326|Merodontini
Eumerus sogdianus|BCSY0331|Merodontini
Eumerus amoenus|BCSY0339|Merodontini
Eumerus strigatus|BCSY0332|Merodontini
Eumerus strigatus|BCSY0333|Merodontini
Eumerus funeralis|BCSY0320|Merodontini
Eumerus sogdianus|BCSY0330|Merodontini
Eumerus vandenberghei|BCSY0341|Merodontini
Merodon constans|BCSY0696|Merodontini
Merodon rufus|BCSY0702|Merodontini
Merodon rufus|BCSY0703|Merodontini
Merodon equestris|BCSY0698|Merodontini
Merodon equestris|BCSY0699|Merodontini
Merodon geniculatus|BCSY0700|Merodontini
Merodon geniculatus|BCSY0701|Merodontini
Merodon albifrons|BCSY0706|Merodontini
Merodon clavipes|BCSY0695|Merodontini
Merodon elegans|BCSY0697|Merodontini
Merodon avidus|BCSY0709|Merodontini
Merodon avidus|BCSY0710|Merodontini
Merodon caucasicus|BCSY0681|Merodontini
Merodon alexeji|BCSY0708|Merodontini
Merodon femoratus|BCSY0711|Merodontini
Merodon aeneus|BCSY0704|Merodontini
Merodon cinereus|BCSY0712|Merodontini
Merodon cinereus|BCSY0713|Merodontini
Callicera spinolae|BCSY0051|Callicerini
Callicera spinolae|BCSY0050|Callicerini
Callicera spinolae|BCSY0052|Callicerini
Eupeodes tirolensis|BCSY0358|Syrphini
Eupeodes nielseni|BCSY0355|Syrphini
Eupeodes nielseni|BCSY0354|Syrphini
Epistrophe nitidicollis|BCSY0289|Syrphini
Eupeodes corollae|BCSY0345|Syrphini
Eupeodes lucasi|BCSY0350|Syrphini
Eupeodes lucasi|BCSY0351|Syrphini
Eupeodes luniger|BCSY0353|Syrphini
Eupeodes tirolensis|BCSY0359|Syrphini
Eupeodes luniger|BCSY0352|Syrphini
Eupeodes flaviceps|BCSY0347|Syrphini
Eupeodes flaviceps|BCSY0346|Syrphini
Eupeodes corollae|BCSY0344|Syrphini
Eupeodes bucculatus|BCSY0343|Syrphini
Eupeodes bucculatus|BCSY0342|Syrphini
Eupeodes latifasciatus|BCSY0348|Syrphini
Eupeodes nitens|BCSY0356|Syrphini
Xanthogramma laetum|BCSY0656|Syrphini
Xanthogramma dives|BCSY0653|Syrphini
Xanthogramma pedissequum|BCSY0659|Syrphini
Eupeodes nitens|BCSY0357|Syrphini
Meligramma cingulata|BCSY0416|Syrphini
Episyrphus balteatus|BCSY0294|Syrphini
Meliscaeva auricollis|BCSY0417|Syrphini
Meliscaeva auricollis|BCSY0418|Syrphini
Meliscaeva cinctella|BCSY0419|Syrphini
Meliscaeva cinctella|BCSY0420|Syrphini
Melangyna barbifrons|BCSY0396|Syrphini
Melangyna barbifrons|BCSY0397|Syrphini
Xanthandrus comtus|BCSY0651|Melanostomini
Xanthandrus comtus|BCSY0652|Melanostomini
Melanostoma certum|BCSY0405|Melanostomini
Melanostoma certum|BCSY0406|Melanostomini
Melanostoma scalare|BCSY0410|Melanostomini
Melangyna lasiophthalma|BCSY0399|Syrphini
Melangyna lasiophthalma|BCSY0400|Syrphini
Melangyna quadrimaculata|BCSY0401|Syrphini
Parasyrphus annulatus|BCSY0477|Syrphini
Parasyrphus annulatus|BCSY0478|Syrphini
Syrphus auberti|BCSY0619|Syrphini
Parasyrphus macularis|BCSY0481|Syrphini
Parasyrphus macularis|BCSY0482|Syrphini
Parasyrphus lineolus|BCSY0479|Syrphini
Parasyrphus lineolus|BCSY0480|Syrphini
Parasyrphus vittiger|BCSY0487|Syrphini
Meligramma cincta|BCSY0413|Syrphini

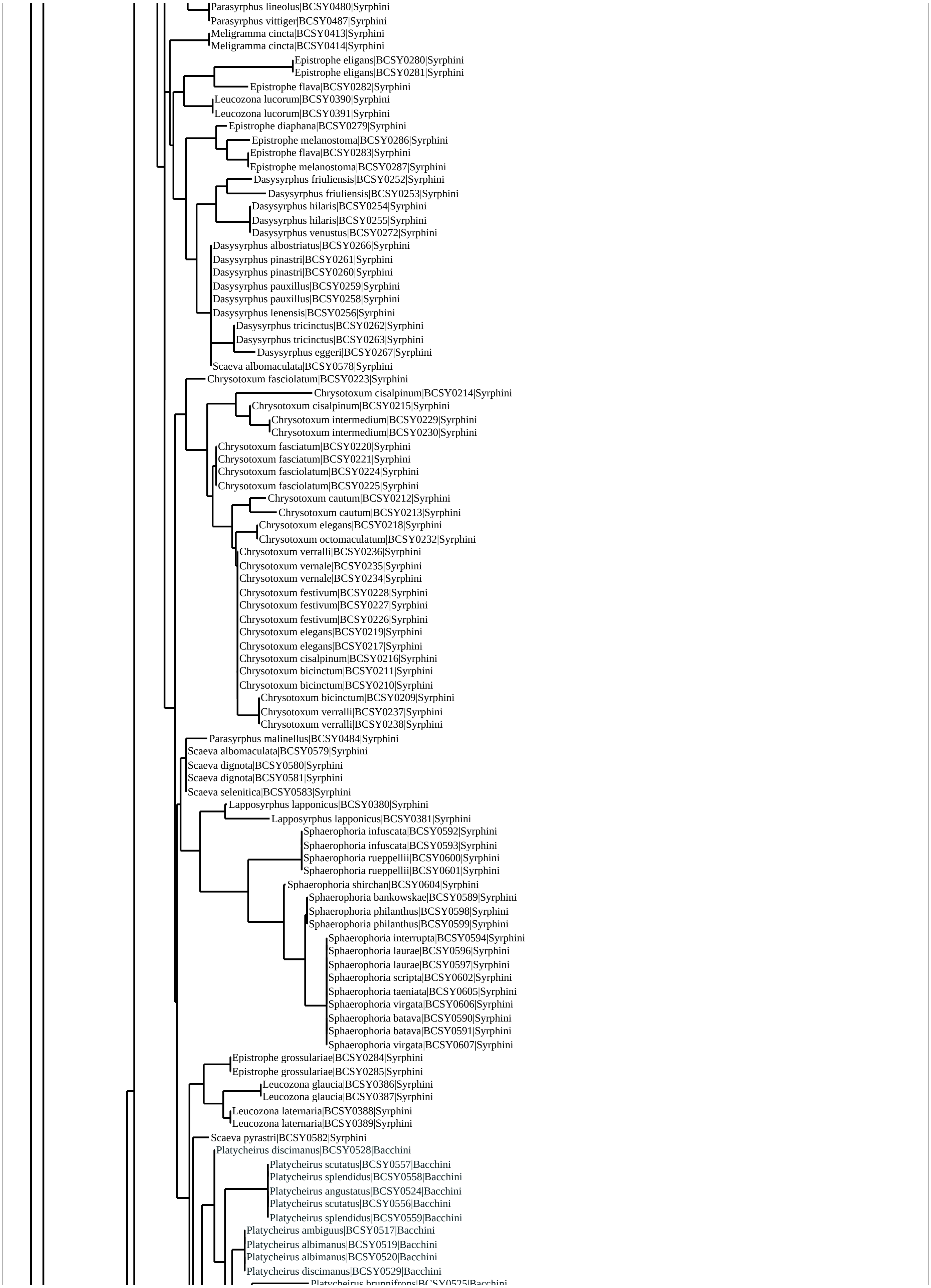
Parasyrphus lineolus|BCSY0480|Syrphini
Parasyrphus vittiger|BCSY0487|Syrphini
Meligramma cincta|BCSY0413|Syrphini
Meligramma cincta|BCSY0414|Syrphini
Epistrophe eligans|BCSY0280|Syrphini
Epistrophe eligans|BCSY0281|Syrphini
Epistrophe flava|BCSY0282|Syrphini
Leucozona lucorum|BCSY0390|Syrphini
Leucozona lucorum|BCSY0391|Syrphini
Epistrophe diaphana|BCSY0279|Syrphini
Epistrophe melanostoma|BCSY0286|Syrphini
Epistrophe flava|BCSY0283|Syrphini
Epistrophe melanostoma|BCSY0287|Syrphini
Dasysyrphus friuliensis|BCSY0252|Syrphini
Dasysyrphus friuliensis|BCSY0253|Syrphini
Dasysyrphus hilaris|BCSY0254|Syrphini
Dasysyrphus hilaris|BCSY0255|Syrphini
Dasysyrphus venustus|BCSY0272|Syrphini
Dasysyrphus albostriatus|BCSY0266|Syrphini
Dasysyrphus pinastri|BCSY0261|Syrphini
Dasysyrphus pinastri|BCSY0260|Syrphini
Dasysyrphus pauxillus|BCSY0259|Syrphini
Dasysyrphus pauxillus|BCSY0258|Syrphini
Dasysyrphus lenensis|BCSY0256|Syrphini
Dasysyrphus tricinctus|BCSY0262|Syrphini
Dasysyrphus tricinctus|BCSY0263|Syrphini
Dasysyrphus eggeri|BCSY0267|Syrphini
Scaeva albomaculata|BCSY0578|Syrphini
Chrysotoxum fasciolatum|BCSY0223|Syrphini
Chrysotoxum cisalpinum|BCSY0214|Syrphini
Chrysotoxum cisalpinum|BCSY0215|Syrphini
Chrysotoxum intermedium|BCSY0229|Syrphini
Chrysotoxum intermedium|BCSY0230|Syrphini
Chrysotoxum fasciatum|BCSY0220|Syrphini
Chrysotoxum fasciatum|BCSY0221|Syrphini
Chrysotoxum fasciolatum|BCSY0224|Syrphini
Chrysotoxum fasciolatum|BCSY0225|Syrphini
Chrysotoxum cautum|BCSY0212|Syrphini
Chrysotoxum cautum|BCSY0213|Syrphini
Chrysotoxum elegans|BCSY0218|Syrphini
Chrysotoxum octomaculatum|BCSY0232|Syrphini
Chrysotoxum verralli|BCSY0236|Syrphini
Chrysotoxum vernale|BCSY0235|Syrphini
Chrysotoxum vernale|BCSY0234|Syrphini
Chrysotoxum festivum|BCSY0228|Syrphini
Chrysotoxum festivum|BCSY0227|Syrphini
Chrysotoxum festivum|BCSY0226|Syrphini
Chrysotoxum elegans|BCSY0219|Syrphini
Chrysotoxum elegans|BCSY0217|Syrphini
Chrysotoxum cisalpinum|BCSY0216|Syrphini
Chrysotoxum bicinctum|BCSY0211|Syrphini
Chrysotoxum bicinctum|BCSY0210|Syrphini
Chrysotoxum bicinctum|BCSY0209|Syrphini
Chrysotoxum verralli|BCSY0237|Syrphini
Chrysotoxum verralli|BCSY0238|Syrphini
Parasyrphus malinellus|BCSY0484|Syrphini
Scaeva albomaculata|BCSY0579|Syrphini
Scaeva dignota|BCSY0580|Syrphini
Scaeva dignota|BCSY0581|Syrphini
Scaeva selenitica|BCSY0583|Syrphini
Lapposyrphus lapponicus|BCSY0380|Syrphini
Lapposyrphus lapponicus|BCSY0381|Syrphini
Sphaerophoria infuscata|BCSY0592|Syrphini
Sphaerophoria infuscata|BCSY0593|Syrphini
Sphaerophoria rueppellii|BCSY0600|Syrphini
Sphaerophoria rueppellii|BCSY0601|Syrphini
Sphaerophoria shirchan|BCSY0604|Syrphini
Sphaerophoria bankowskae|BCSY0589|Syrphini
Sphaerophoria philanthus|BCSY0598|Syrphini
Sphaerophoria philanthus|BCSY0599|Syrphini
Sphaerophoria interrupta|BCSY0594|Syrphini
Sphaerophoria laurae|BCSY0596|Syrphini
Sphaerophoria laurae|BCSY0597|Syrphini
Sphaerophoria scripta|BCSY0602|Syrphini
Sphaerophoria taeniata|BCSY0605|Syrphini
Sphaerophoria virgata|BCSY0606|Syrphini
Sphaerophoria batava|BCSY0590|Syrphini
Sphaerophoria batava|BCSY0591|Syrphini
Sphaerophoria virgata|BCSY0607|Syrphini
Epistrophe grossulariae|BCSY0284|Syrphini
Epistrophe grossulariae|BCSY0285|Syrphini
Leucozona glaucia|BCSY0386|Syrphini
Leucozona glaucia|BCSY0387|Syrphini
Leucozona laternaria|BCSY0388|Syrphini
Leucozona laternaria|BCSY0389|Syrphini
Scaeva pyrastri|BCSY0582|Syrphini
Platycheirus discimanus|BCSY0528|Bacchini
Platycheirus scutatus|BCSY0557|Bacchini
Platycheirus splendidus|BCSY0558|Bacchini
Platycheirus angustatus|BCSY0524|Bacchini
Platycheirus scutatus|BCSY0556|Bacchini
Platycheirus splendidus|BCSY0559|Bacchini
Platycheirus ambiguus|BCSY0517|Bacchini
Platycheirus albimanus|BCSY0519|Bacchini
Platycheirus albimanus|BCSY0520|Bacchini
Platycheirus discimanus|BCSY0529|Bacchini
Platycheirus brunnifrons|BCSY0525|Bacchini

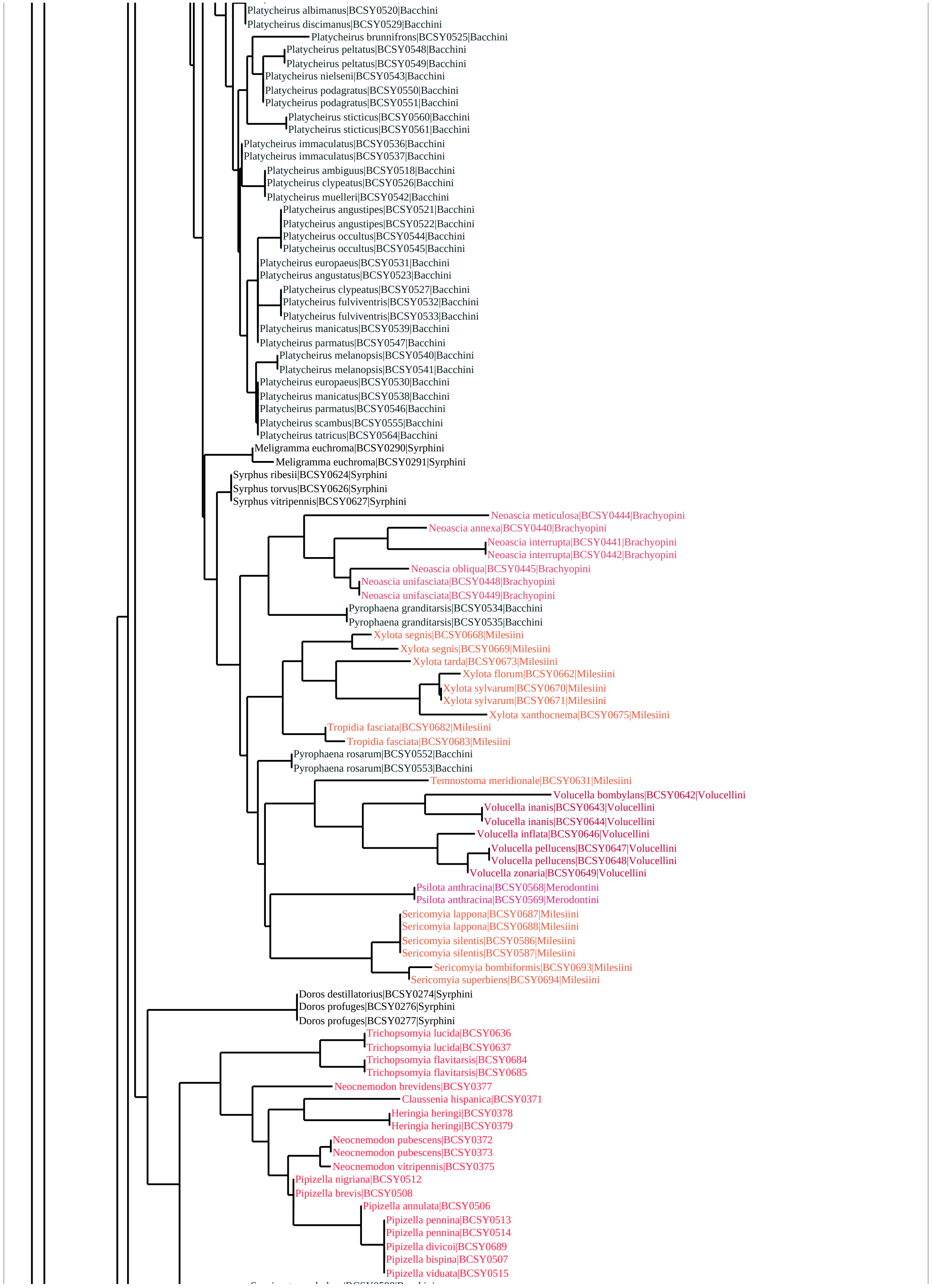
Platycheirus albimanus|BCSY0520|Bacchini
Platycheirus discimanus|BCSY0529|Bacchini
Platycheirus brunnifrons|BCSY0525|Bacchini
Platycheirus peltatus|BCSY0548|Bacchini
Platycheirus peltatus|BCSY0549|Bacchini
Platycheirus nielseni|BCSY0543|Bacchini
Platycheirus podagratus|BCSY0550|Bacchini
Platycheirus podagratus|BCSY0551|Bacchini
Platycheirus sticticus|BCSY0560|Bacchini
Platycheirus sticticus|BCSY0561|Bacchini
Platycheirus immaculatus|BCSY0536|Bacchini
Platycheirus immaculatus|BCSY0537|Bacchini
Platycheirus ambiguus|BCSY0518|Bacchini
Platycheirus clypeatus|BCSY0526|Bacchini
Platycheirus muelleri|BCSY0542|Bacchini
Platycheirus angustipes|BCSY0521|Bacchini
Platycheirus angustipes|BCSY0522|Bacchini
Platycheirus occultus|BCSY0544|Bacchini
Platycheirus occultus|BCSY0545|Bacchini
Platycheirus europaeus|BCSY0531|Bacchini
Platycheirus angustatus|BCSY0523|Bacchini
Platycheirus clypeatus|BCSY0527|Bacchini
Platycheirus fulviventris|BCSY0532|Bacchini
Platycheirus fulviventris|BCSY0533|Bacchini
Platycheirus manicatus|BCSY0539|Bacchini
Platycheirus parmatus|BCSY0547|Bacchini
Platycheirus melanopsis|BCSY0540|Bacchini
Platycheirus melanopsis|BCSY0541|Bacchini
Platycheirus europaeus|BCSY0530|Bacchini
Platycheirus manicatus|BCSY0538|Bacchini
Platycheirus parmatus|BCSY0546|Bacchini
Platycheirus scambus|BCSY0555|Bacchini
Platycheirus tatricus|BCSY0564|Bacchini
Meligramma euchroma|BCSY0290|Syrphini
Meligramma euchroma|BCSY0291|Syrphini
Syrphus ribesii|BCSY0624|Syrphini
Syrphus torvus|BCSY0626|Syrphini
Syrphus vitripennis|BCSY0627|Syrphini
Neoascia meticulosa|BCSY0444|Brachyopini
Neoascia annexa|BCSY0440|Brachyopini
Neoascia interrupta|BCSY0441|Brachyopini
Neoascia interrupta|BCSY0442|Brachyopini
Neoascia obliqua|BCSY0445|Brachyopini
Neoascia unifasciata|BCSY0448|Brachyopini
Neoascia unifasciata|BCSY0449|Brachyopini
Pyrophaena granditarsis|BCSY0534|Bacchini
Pyrophaena granditarsis|BCSY0535|Bacchini
Xylota segnis|BCSY0668|Milesiini
Xylota segnis|BCSY0669|Milesiini
Xylota tarda|BCSY0673|Milesiini
Xylota florum|BCSY0662|Milesiini
Xylota sylvarum|BCSY0670|Milesiini
Xylota sylvarum|BCSY0671|Milesiini
Xylota xanthocnema|BCSY0675|Milesiini
Tropidia fasciata|BCSY0682|Milesiini
Tropidia fasciata|BCSY0683|Milesiini
Pyrophaena rosarum|BCSY0552|Bacchini
Pyrophaena rosarum|BCSY0553|Bacchini
Temnostoma meridionale|BCSY0631|Milesiini
Volucella bombylans|BCSY0642|Volucellini
Volucella inanis|BCSY0643|Volucellini
Volucella inanis|BCSY0644|Volucellini
Volucella inflata|BCSY0646|Volucellini
Volucella pellucens|BCSY0647|Volucellini
Volucella pellucens|BCSY0648|Volucellini
Volucella zonaria|BCSY0649|Volucellini
Psilota anthracina|BCSY0568|Merodontini
Psilota anthracina|BCSY0569|Merodontini
Sericomyia lappona|BCSY0687|Milesiini
Sericomyia lappona|BCSY0688|Milesiini
Sericomyia silentis|BCSY0586|Milesiini
Sericomyia silentis|BCSY0587|Milesiini
Sericomyia bombiformis|BCSY0693|Milesiini
Sericomyia superbiens|BCSY0694|Milesiini
Doros destillatorius|BCSY0274|Syrphini
Doros profuges|BCSY0276|Syrphini
Doros profuges|BCSY0277|Syrphini
Trichopsomyia lucida|BCSY0636
Trichopsomyia lucida|BCSY0637
Trichopsomyia flavitarsis|BCSY0684
Trichopsomyia flavitarsis|BCSY0685
Neocnemodon brevidens|BCSY0377
Claussenia hispanica|BCSY0371
Heringia heringi|BCSY0378
Heringia heringi|BCSY0379
Neocnemodon pubescens|BCSY0372
Neocnemodon pubescens|BCSY0373
Neocnemodon vitripennis|BCSY0375
Pipizella nigriana|BCSY0512
Pipizella brevis|BCSY0508
Pipizella annulata|BCSY0506
Pipizella pennina|BCSY0513
Pipizella pennina|BCSY0514
Pipizella divicoi|BCSY0689
Pipizella bispina|BCSY0507
Pipizella viduata|BCSY0515

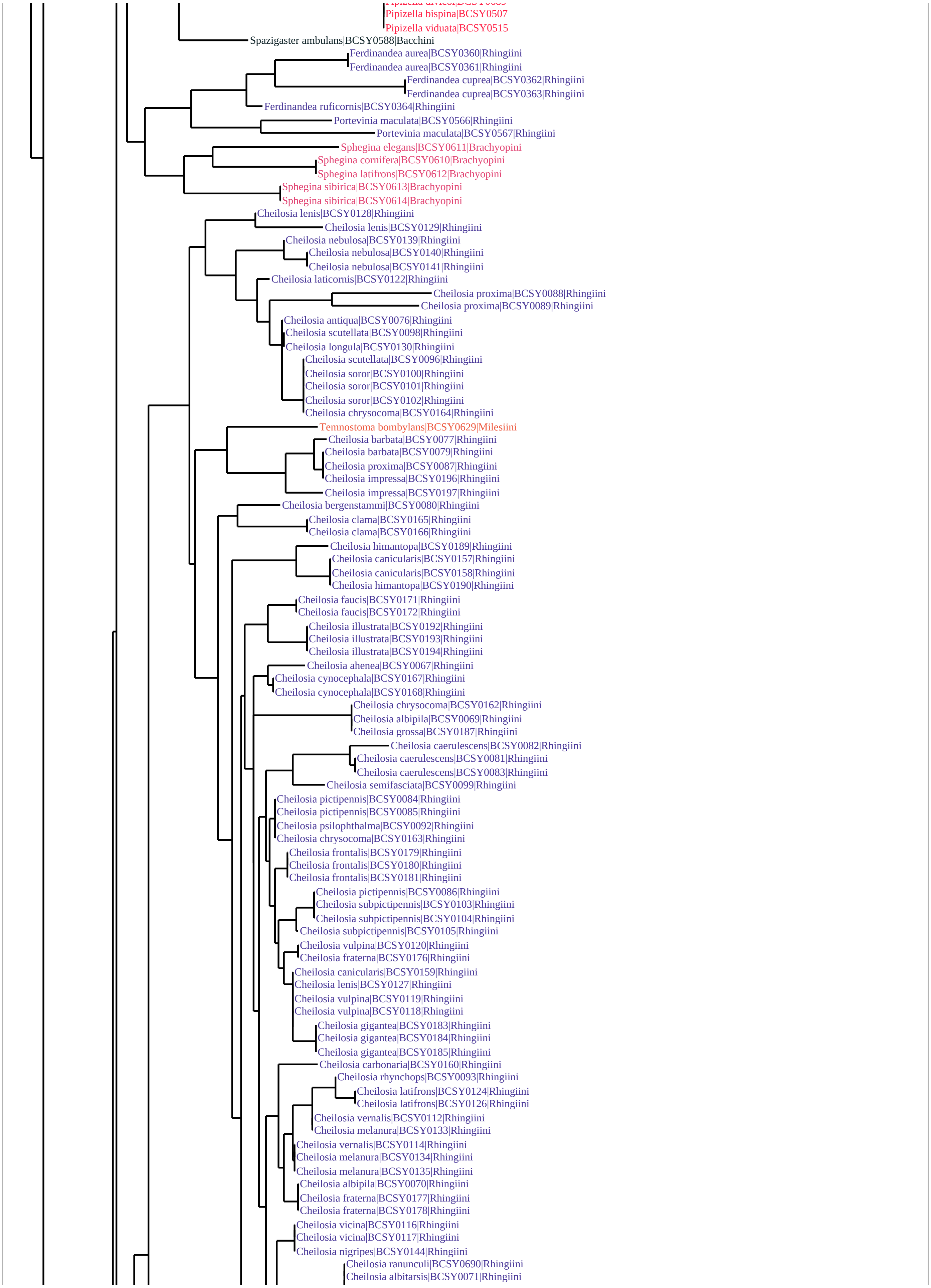
Pipizella bispina|BCSY0507
Pipizella viduata|BCSY0515
Spazigaster ambulans|BCSY0588|Bacchini
Ferdinandea aurea|BCSY0360|Rhingiini
Ferdinandea aurea|BCSY0361|Rhingiini
Ferdinandea cuprea|BCSY0362|Rhingiini
Ferdinandea cuprea|BCSY0363|Rhingiini
Ferdinandea ruficornis|BCSY0364|Rhingiini
Portevinia maculata|BCSY0566|Rhingiini
Portevinia maculata|BCSY0567|Rhingiini
Sphegina elegans|BCSY0611|Brachyopini
Sphegina cornifera|BCSY0610|Brachyopini
Sphegina latifrons|BCSY0612|Brachyopini
Sphegina sibirica|BCSY0613|Brachyopini
Sphegina sibirica|BCSY0614|Brachyopini
Cheilosia lenis|BCSY0128|Rhingiini
Cheilosia lenis|BCSY0129|Rhingiini
Cheilosia nebulosa|BCSY0139|Rhingiini
Cheilosia nebulosa|BCSY0140|Rhingiini
Cheilosia nebulosa|BCSY0141|Rhingiini
Cheilosia laticornis|BCSY0122|Rhingiini
Cheilosia proxima|BCSY0088|Rhingiini
Cheilosia proxima|BCSY0089|Rhingiini
Cheilosia antiqua|BCSY0076|Rhingiini
Cheilosia scutellata|BCSY0098|Rhingiini
Cheilosia longula|BCSY0130|Rhingiini
Cheilosia scutellata|BCSY0096|Rhingiini
Cheilosia soror|BCSY0100|Rhingiini
Cheilosia soror|BCSY0101|Rhingiini
Cheilosia soror|BCSY0102|Rhingiini
Cheilosia chrysocoma|BCSY0164|Rhingiini
Temnostoma bombylans|BCSY0629|Milesiini
Cheilosia barbata|BCSY0077|Rhingiini
Cheilosia barbata|BCSY0079|Rhingiini
Cheilosia proxima|BCSY0087|Rhingiini
Cheilosia impressa|BCSY0196|Rhingiini
Cheilosia impressa|BCSY0197|Rhingiini
Cheilosia bergenstammi|BCSY0080|Rhingiini
Cheilosia clama|BCSY0165|Rhingiini
Cheilosia clama|BCSY0166|Rhingiini
Cheilosia himantopa|BCSY0189|Rhingiini
Cheilosia canicularis|BCSY0157|Rhingiini
Cheilosia canicularis|BCSY0158|Rhingiini
Cheilosia himantopa|BCSY0190|Rhingiini
Cheilosia faucis|BCSY0171|Rhingiini
Cheilosia faucis|BCSY0172|Rhingiini
Cheilosia illustrata|BCSY0192|Rhingiini
Cheilosia illustrata|BCSY0193|Rhingiini
Cheilosia illustrata|BCSY0194|Rhingiini
Cheilosia ahenea|BCSY0067|Rhingiini
Cheilosia cynocephala|BCSY0167|Rhingiini
Cheilosia cynocephala|BCSY0168|Rhingiini
Cheilosia chrysocoma|BCSY0162|Rhingiini
Cheilosia albipila|BCSY0069|Rhingiini
Cheilosia grossa|BCSY0187|Rhingiini
Cheilosia caerulescens|BCSY0082|Rhingiini
Cheilosia caerulescens|BCSY0081|Rhingiini
Cheilosia caerulescens|BCSY0083|Rhingiini
Cheilosia semifasciata|BCSY0099|Rhingiini
Cheilosia pictipennis|BCSY0084|Rhingiini
Cheilosia pictipennis|BCSY0085|Rhingiini
Cheilosia psilophthalma|BCSY0092|Rhingiini
Cheilosia chrysocoma|BCSY0163|Rhingiini
Cheilosia frontalis|BCSY0179|Rhingiini
Cheilosia frontalis|BCSY0180|Rhingiini
Cheilosia frontalis|BCSY0181|Rhingiini
Cheilosia pictipennis|BCSY0086|Rhingiini
Cheilosia subpictipennis|BCSY0103|Rhingiini
Cheilosia subpictipennis|BCSY0104|Rhingiini
Cheilosia subpictipennis|BCSY0105|Rhingiini
Cheilosia vulpina|BCSY0120|Rhingiini
Cheilosia fraterna|BCSY0176|Rhingiini
Cheilosia canicularis|BCSY0159|Rhingiini
Cheilosia lenis|BCSY0127|Rhingiini
Cheilosia vulpina|BCSY0119|Rhingiini
Cheilosia vulpina|BCSY0118|Rhingiini
Cheilosia gigantea|BCSY0183|Rhingiini
Cheilosia gigantea|BCSY0184|Rhingiini
Cheilosia gigantea|BCSY0185|Rhingiini
Cheilosia carbonaria|BCSY0160|Rhingiini
Cheilosia rhynchops|BCSY0093|Rhingiini
Cheilosia latifrons|BCSY0124|Rhingiini
Cheilosia latifrons|BCSY0126|Rhingiini
Cheilosia vernalis|BCSY0112|Rhingiini
Cheilosia melanura|BCSY0133|Rhingiini
Cheilosia vernalis|BCSY0114|Rhingiini
Cheilosia melanura|BCSY0134|Rhingiini
Cheilosia melanura|BCSY0135|Rhingiini
Cheilosia albipila|BCSY0070|Rhingiini
Cheilosia fraterna|BCSY0177|Rhingiini
Cheilosia fraterna|BCSY0178|Rhingiini
Cheilosia vicina|BCSY0116|Rhingiini
Cheilosia vicina|BCSY0117|Rhingiini
Cheilosia nigripes|BCSY0144|Rhingiini
Cheilosia ranunculi|BCSY0690|Rhingiini
Cheilosia albitarsis|BCSY0071|Rhingiini

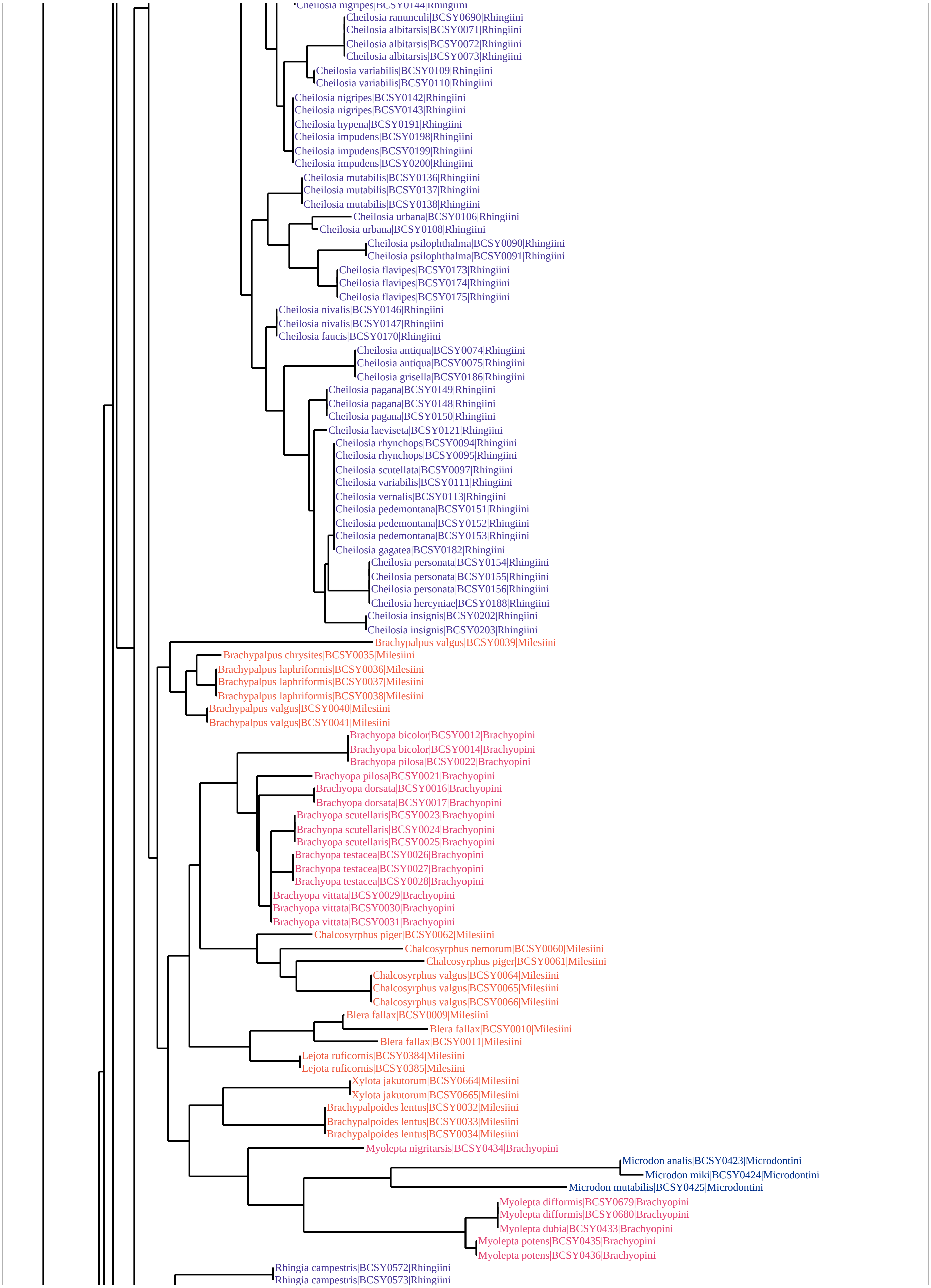
Cheilosia nigripes|BCSY0144|Rhingiini
Cheilosia ranunculi|BCSY0690|Rhingiini
Cheilosia albitarsis|BCSY0071|Rhingiini
Cheilosia albitarsis|BCSY0072|Rhingiini
Cheilosia albitarsis|BCSY0073|Rhingiini
Cheilosia variabilis|BCSY0109|Rhingiini
Cheilosia variabilis|BCSY0110|Rhingiini
Cheilosia nigripes|BCSY0142|Rhingiini
Cheilosia nigripes|BCSY0143|Rhingiini
Cheilosia hypena|BCSY0191|Rhingiini
Cheilosia impudens|BCSY0198|Rhingiini
Cheilosia impudens|BCSY0199|Rhingiini
Cheilosia impudens|BCSY0200|Rhingiini
Cheilosia mutabilis|BCSY0136|Rhingiini
Cheilosia mutabilis|BCSY0137|Rhingiini
Cheilosia mutabilis|BCSY0138|Rhingiini
Cheilosia urbana|BCSY0106|Rhingiini
Cheilosia urbana|BCSY0108|Rhingiini
Cheilosia psilophthalma|BCSY0090|Rhingiini
Cheilosia psilophthalma|BCSY0091|Rhingiini
Cheilosia flavipes|BCSY0173|Rhingiini
Cheilosia flavipes|BCSY0174|Rhingiini
Cheilosia flavipes|BCSY0175|Rhingiini
Cheilosia nivalis|BCSY0146|Rhingiini
Cheilosia nivalis|BCSY0147|Rhingiini
Cheilosia faucis|BCSY0170|Rhingiini
Cheilosia antiqua|BCSY0074|Rhingiini
Cheilosia antiqua|BCSY0075|Rhingiini
Cheilosia grisella|BCSY0186|Rhingiini
Cheilosia pagana|BCSY0149|Rhingiini
Cheilosia pagana|BCSY0148|Rhingiini
Cheilosia pagana|BCSY0150|Rhingiini
Cheilosia laeviseta|BCSY0121|Rhingiini
Cheilosia rhynchops|BCSY0094|Rhingiini
Cheilosia rhynchops|BCSY0095|Rhingiini
Cheilosia scutellata|BCSY0097|Rhingiini
Cheilosia variabilis|BCSY0111|Rhingiini
Cheilosia vernalis|BCSY0113|Rhingiini
Cheilosia pedemontana|BCSY0151|Rhingiini
Cheilosia pedemontana|BCSY0152|Rhingiini
Cheilosia pedemontana|BCSY0153|Rhingiini
Cheilosia gagatea|BCSY0182|Rhingiini
Cheilosia personata|BCSY0154|Rhingiini
Cheilosia personata|BCSY0155|Rhingiini
Cheilosia personata|BCSY0156|Rhingiini
Cheilosia hercyniae|BCSY0188|Rhingiini
Cheilosia insignis|BCSY0202|Rhingiini
Cheilosia insignis|BCSY0203|Rhingiini
Brachypalpus valgus|BCSY0039|Milesiini
Brachypalpus chrysites|BCSY0035|Milesiini
Brachypalpus laphriformis|BCSY0036|Milesiini
Brachypalpus laphriformis|BCSY0037|Milesiini
Brachypalpus laphriformis|BCSY0038|Milesiini
Brachypalpus valgus|BCSY0040|Milesiini
Brachypalpus valgus|BCSY0041|Milesiini
Brachyopa bicolor|BCSY0012|Brachyopini
Brachyopa bicolor|BCSY0014|Brachyopini
Brachyopa pilosa|BCSY0022|Brachyopini
Brachyopa pilosa|BCSY0021|Brachyopini
Brachyopa dorsata|BCSY0016|Brachyopini
Brachyopa dorsata|BCSY0017|Brachyopini
Brachyopa scutellaris|BCSY0023|Brachyopini
Brachyopa scutellaris|BCSY0024|Brachyopini
Brachyopa scutellaris|BCSY0025|Brachyopini
Brachyopa testacea|BCSY0026|Brachyopini
Brachyopa testacea|BCSY0027|Brachyopini
Brachyopa testacea|BCSY0028|Brachyopini
Brachyopa vittata|BCSY0029|Brachyopini
Brachyopa vittata|BCSY0030|Brachyopini
Brachyopa vittata|BCSY0031|Brachyopini
Chalcosyrphus piger|BCSY0062|Milesiini
Chalcosyrphus nemorum|BCSY0060|Milesiini
Chalcosyrphus piger|BCSY0061|Milesiini
Chalcosyrphus valgus|BCSY0064|Milesiini
Chalcosyrphus valgus|BCSY0065|Milesiini
Chalcosyrphus valgus|BCSY0066|Milesiini
Blera fallax|BCSY0009|Milesiini
Blera fallax|BCSY0010|Milesiini
Blera fallax|BCSY0011|Milesiini
Lejota ruficornis|BCSY0384|Milesiini
Lejota ruficornis|BCSY0385|Milesiini
Xylota jakutorum|BCSY0664|Milesiini
Xylota jakutorum|BCSY0665|Milesiini
Brachypalpoides lentus|BCSY0032|Milesiini
Brachypalpoides lentus|BCSY0033|Milesiini
Brachypalpoides lentus|BCSY0034|Milesiini
Myolepta nigritarsis|BCSY0434|Brachyopini
Microdon analis|BCSY0423|Microdontini
Microdon miki|BCSY0424|Microdontini
Microdon mutabilis|BCSY0425|Microdontini
Myolepta difformis|BCSY0679|Brachyopini
Myolepta difformis|BCSY0680|Brachyopini
Myolepta dubia|BCSY0433|Brachyopini
Myolepta potens|BCSY0435|Brachyopini
Myolepta potens|BCSY0436|Brachyopini
Rhingia campestris|BCSY0572|Rhingiini
Rhingia campestris|BCSY0573|Rhingiini

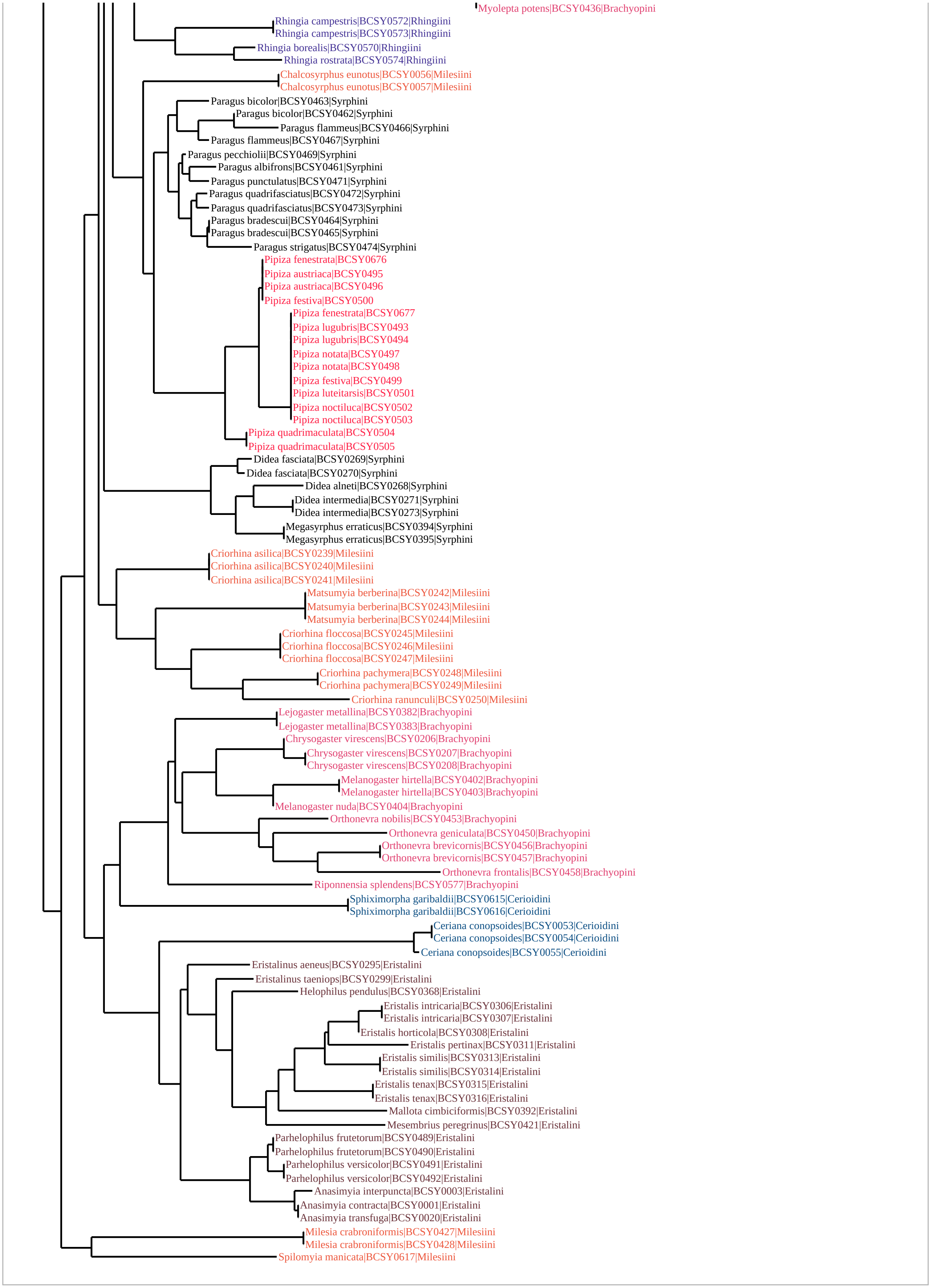
Myolepta potens|BCSY0436|Brachyopini
Rhingia campestris|BCSY0572|Rhingiini
Rhingia campestris|BCSY0573|Rhingiini
Rhingia borealis|BCSY0570|Rhingiini
Rhingia rostrata|BCSY0574|Rhingiini
Chalcosyrphus eunotus|BCSY0056|Milesiini
Chalcosyrphus eunotus|BCSY0057|Milesiini
Paragus bicolor|BCSY0463|Syrphini
Paragus bicolor|BCSY0462|Syrphini
Paragus flammeus|BCSY0466|Syrphini
Paragus flammeus|BCSY0467|Syrphini
Paragus pecchiolii|BCSY0469|Syrphini
Paragus albifrons|BCSY0461|Syrphini
Paragus punctulatus|BCSY0471|Syrphini
Paragus quadrifasciatus|BCSY0472|Syrphini
Paragus quadrifasciatus|BCSY0473|Syrphini
Paragus bradescui|BCSY0464|Syrphini
Paragus bradescui|BCSY0465|Syrphini
Paragus strigatus|BCSY0474|Syrphini
Pipiza fenestrata|BCSY0676
Pipiza austriaca|BCSY0495
Pipiza austriaca|BCSY0496
Pipiza festiva|BCSY0500
Pipiza fenestrata|BCSY0677
Pipiza lugubris|BCSY0493
Pipiza lugubris|BCSY0494
Pipiza notata|BCSY0497
Pipiza notata|BCSY0498
Pipiza festiva|BCSY0499
Pipiza luteitarsis|BCSY0501
Pipiza noctiluca|BCSY0502
Pipiza noctiluca|BCSY0503
Pipiza quadrimaculata|BCSY0504
Pipiza quadrimaculata|BCSY0505
Didea fasciata|BCSY0269|Syrphini
Didea fasciata|BCSY0270|Syrphini
Didea alneti|BCSY0268|Syrphini
Didea intermedia|BCSY0271|Syrphini
Didea intermedia|BCSY0273|Syrphini
Megasyrphus erraticus|BCSY0394|Syrphini
Megasyrphus erraticus|BCSY0395|Syrphini
Criorhina asilica|BCSY0239|Milesiini
Criorhina asilica|BCSY0240|Milesiini
Criorhina asilica|BCSY0241|Milesiini
Matsumyia berberina|BCSY0242|Milesiini
Matsumyia berberina|BCSY0243|Milesiini
Matsumyia berberina|BCSY0244|Milesiini
Criorhina floccosa|BCSY0245|Milesiini
Criorhina floccosa|BCSY0246|Milesiini
Criorhina floccosa|BCSY0247|Milesiini
Criorhina pachymera|BCSY0248|Milesiini
Criorhina pachymera|BCSY0249|Milesiini
Criorhina ranunculi|BCSY0250|Milesiini
Lejogaster metallina|BCSY0382|Brachyopini
Lejogaster metallina|BCSY0383|Brachyopini
Chrysogaster virescens|BCSY0206|Brachyopini
Chrysogaster virescens|BCSY0207|Brachyopini
Chrysogaster virescens|BCSY0208|Brachyopini
Melanogaster hirtella|BCSY0402|Brachyopini
Melanogaster hirtella|BCSY0403|Brachyopini
Melanogaster nuda|BCSY0404|Brachyopini
Orthonevra nobilis|BCSY0453|Brachyopini
Orthonevra geniculata|BCSY0450|Brachyopini
Orthonevra brevicornis|BCSY0456|Brachyopini
Orthonevra brevicornis|BCSY0457|Brachyopini
Orthonevra frontalis|BCSY0458|Brachyopini
Riponnensia splendens|BCSY0577|Brachyopini
Sphiximorpha garibaldii|BCSY0615|Cerioidini
Sphiximorpha garibaldii|BCSY0616|Cerioidini
Ceriana conopsoides|BCSY0053|Cerioidini
Ceriana conopsoides|BCSY0054|Cerioidini
Ceriana conopsoides|BCSY0055|Cerioidini
Eristalinus aeneus|BCSY0295|Eristalini
Eristalinus taeniops|BCSY0299|Eristalini
Helophilus pendulus|BCSY0368|Eristalini
Eristalis intricaria|BCSY0306|Eristalini
Eristalis intricaria|BCSY0307|Eristalini
Eristalis horticola|BCSY0308|Eristalini
Eristalis pertinax|BCSY0311|Eristalini
Eristalis similis|BCSY0313|Eristalini
Eristalis similis|BCSY0314|Eristalini
Eristalis tenax|BCSY0315|Eristalini
Eristalis tenax|BCSY0316|Eristalini
Mallota cimbiciformis|BCSY0392|Eristalini
Mesembrius peregrinus|BCSY0421|Eristalini
Parhelophilus frutetorum|BCSY0489|Eristalini
Parhelophilus frutetorum|BCSY0490|Eristalini
Parhelophilus versicolor|BCSY0491|Eristalini
Parhelophilus versicolor|BCSY0492|Eristalini
Anasimyia interpuncta|BCSY0003|Eristalini
Anasimyia contracta|BCSY0001|Eristalini
Anasimyia transfuga|BCSY0020|Eristalini
Milesia crabroniformis|BCSY0427|Milesiini
Milesia crabroniformis|BCSY0428|Milesiini
Spilomyia manicata|BCSY0617|Milesiini
